# A proof-of-concept study of an albumin-based bilayered scaffold for cartilage regeneration

**DOI:** 10.1016/j.mtbio.2026.103193

**Published:** 2026-05-02

**Authors:** Christelle Bertsch, Florent Colin, Eya Aloui, Julien Graff, Maria Cristina Antal, Sabine Kuchler-Bopp, Adrien Moya, Romy Marek, Sven Zaugg, Eric Mathieu, Claire Thibault, Christian Debry, Jordan Beurton, Bernard Senger, Benoit Frisch, Michael de Wild, Arnaud Scherberich, Philippe Lavalle, Léa Fath

**Affiliations:** a Inserm UMR_S 1121, CNRS EMR 7003, Université de Strasbourg, Biomaterials and Bioengineering, Centre de Recherche en Biomédecine de Strasbourg, 1 Rue Eugène Boeckel, Strasbourg, F-67000, France; b ALBUPAD SAS, 32 Allée de la Robertsau, Strasbourg, 67000, France; c Institut d’histologie, Faculté de médecine, Université de Strasbourg, Strasbourg, France; d Department of Biomedicine, University of Basel, University Hospital of Basel, Hebelstrasse 20, Basel, 4031, Switzerland; e University of Applied Sciences Northwestern Switzerland FHNW, School of Life Sciences HLS, Institute for Medical Engineering and Medical Informatics IM2, Hofackerstrasse 30, Muttenz, 4132, Switzerland; f Service d’ORL et de Chirurgie Cervico-Faciale, Hôpitaux Universitaires de Strasbourg, 1 Avenue Molière, Strasbourg, 67200, France

**Keywords:** Bilayered scaffolds, Biomaterials, Tissue engineering, Biomimetism, Albumin, Medical applications, Otorhinolaryngology

## Abstract

Cartilage, particularly hyaline cartilage, is essential for structural and functional integrity in otorhinolaryngological region (nose, ears, larynx, and trachea) but exhibits limited regenerative capacity due to its avascular nature. Current clinical strategies, including microfracture, autologous chondrocyte implantation, and cartilage grafting remain limited by poor integration, donor site morbidity, and fibrocartilage formation rather than hyaline cartilage. In parallel, most clinically investigated scaffolds rely on xenogeneic collagen, raising concerns regarding batch-to-batch variability, immunogenicity, regulatory burden, and sourcing. Together, these limitations highlight the need for more clinically translatable autologous, biomimetic, and scalable biomaterials. Here, we report a proof-of-concept albumin-based bilayered scaffold for cartilage tissue engineering, using a salt-assisted compaction process without the use of chemical crosslinkers, based on albumin self-assembly. This scaffold combines a porous layer to support cell infiltration and cartilage-like formation, and a smooth layer to support later the regeneration of cutaneous (auricular) or respiratory (tracheal or nasal) epithelium in situ. In this study, human nasal chondrocytes seeded in the scaffold showed proliferation, maintained viability and were associated with the production of cartilage-like extracellular matrix rich in type II collagen and aggrecan. Following subcutaneous implantation in nude mice, the scaffold showed progressive degradation, tissue integration, and features consistent with hyaline-like cartilage formation. Overall, this work suggests that albumin-based bilayered scaffolds may represent a promising approach for cartilage repair and may be advantageous for applications in nasal, auricular, and craniofacial reconstruction. Further studies in orthotopic models will be required to evaluate their functional performance and clinical relevance.

## Highlights

• A new albumin-based bilayered scaffold fabricated from native albumin without chemical crosslinkers.
• Supporting 3D chondrocyte growth and cartilage-like tissue formation *in vitro* and *in vivo*.
• A biomaterial designed for cartilage tissue engineering applications in otorhinolaryngology, for example.

## Graphical abstract

**Figure:**
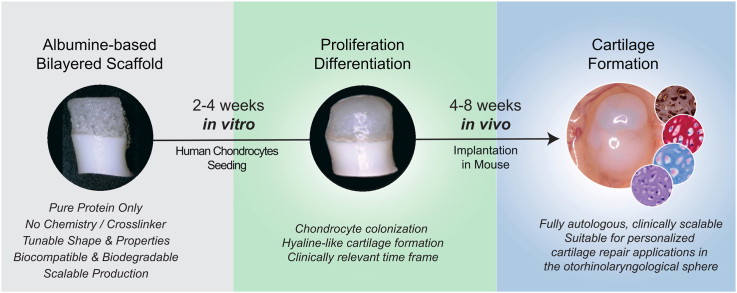

## 1 Introduction

Cartilage is a compact, avascular, and aneural connective tissue embedded in a dense extracellular matrix (ECM) containing sparsely distributed chondrocytes. The ECM is rich in collagen type II, proteoglycans, and sulfated glycosaminoglycans (sGAGs), which provide cartilage with the required mechanical properties for proper *in vivo* function. Cartilage is a flexible tissue that resists compressive forces, maintains structural integrity, and provides support in specific bone areas. However, cartilage has a limited capacity for self-regeneration following damage, making its regeneration a significant challenge [1]. Several strategies have been used to regenerate it, including microfracture [2], autologous chondrocyte transplantation [3,4], cartilage autografts, and allografts [5]. However, these strategies present several notable limitations and drawbacks that can hinder their long-term clinical application: insufficient host integration, donor site morbidity, infection, graft rejection, and, above all, undesirable fibrocartilage regeneration. To date, no engineered substitute has fully replicated the intricate composition and functional properties of cartilage [6]. New approaches suggest that cartilage tissue engineering (TE) techniques, which combine cells and scaffolds, can overcome these limitations. The core principle of cartilage TE is to create a cell-scaffold construct and implant it into the damaged area to restore tissues and/or organs [7]. Cartilage TE aims to generate new cartilage with the correct structure and properties to ensure integration within the patient's tissues. Scaffolds for cartilage regeneration should: i) provide an appropriate environment to support cell proliferation, ii) promote cell differentiation and neocartilage formation through the synthesis of a chondral matrix rich in type II collagen and aggrecan, iii) provide adequate mechanical properties, such as tensile strength, compressive modulus, and elasticity, iv) possess adequate porosity to allow for nutrient and waste exchanges, and v) be biocompatible and biodegradable.

In general, cartilage TE requires a three-dimensional (3D) matrix to promote effective cartilage regeneration [8]. Bidimensional (2D) culture expansion of chondrocytes induces dedifferentiation and a loss of the specific protein synthesis required for cartilage matrix formation [9,10]. Chondrocytes progressively lose their hyaline cartilage phenotype, characterized by the expression of type II collagen and aggrecan, and acquire a fibroblast-like phenotype marked by type I collagen [11,12]. In contrast, a 3D scaffold helps to prevent the dedifferentiation of chondrocytes into fibroblast-like cells. It provides a microenvironment that supports appropriate cell–matrix interactions and promotes the maintenance of the chondrogenic phenotype. To create 3D scaffolds, suitable materials include both natural and synthetic polymers [9,13], as well as their combinations [7]. However, non-biomimetic synthetic materials have unfavorable properties for cartilage regeneration. Indeed, most of them exhibit low biocompatibility and bioactivity, induce aseptic inflammation, and fail to promote ECM regeneration [14]. Conversely, natural materials, particularly proteins, have gained significant attention in TE due to their excellent biocompatibility and biodegradability, low immunogenicity, and broad availability at a low cost [15]. To date, a wide range of natural biopolymers has been considered, including proteins such as albumin [[15], [16], [17]], collagen [18,19], silk fibroin [[20], [21], [22]], and fibrin [23,24], as well as polysaccharides such as starch [25], cellulose [[26], [27], [28]], chitin/chitosan [[29], [30], [31]], and hyaluronic acid [[32], [33], [34]]. Collagen is widely considered as the gold standard for cartilage TE [35]. Significant progress has been made by Martin’s group, which has addressed many of these challenges using the CHONDRO-GIDE® porcine collagen membrane technology [36,37]. In their first-in-human observational trial, Mumme et al. (2016) demonstrated the potential of this bilayered membrane, composed of type I and type III porcine collagen, for cartilage regeneration [38].

The design consists of a porous layer that supports cell infiltration and a compact layer that provides mechanical stability and acts as a barrier to prevent cell leakage. However, most commercially available collagen-based scaffolds intended for clinical applications are typically derived from bovine or other non-human sources, raising concerns about their consistency and positioning them as still improvable xenogeneic solutions. For this reason, and due to its excellent potential for TE, extensive studies have been conducted on albumin [39]. Albumin is the most abundant protein in plasma, where it plays a crucial metabolic role by regulating oncotic pressure and binding to and transporting various molecules, including hormones, enzymes, drugs, and toxins. Albumin is sustainable, cost-effective, and can be sourced allogeneically, or even autologously [[40], [41], [42]]. This makes the development of personalized albumin-based scaffolds for TE highly appealing. Moreover, therapeutic human albumin is already available in pharmaceutical-grade formulations, such as Albunorm, which is commonly used for infusion. To date, research on albumin has primarily focused on developing therapeutic molecules or drug vectors. However, its use as a scaffold for TE has not yet been well established [18]. There have been reports of its successful use as a biomaterial in the form of coatings, hydrogels [15], or scaffolds [43], but few studies have focused on its use as a matrix support for cartilage regeneration [44]. Moreover, the strategies used involved chemical protein modifications, as well as the use of cross-linkers and solvents, which raise potential biosafety concerns.

We recently developed albumin-based materials using salt-assisted compaction, based on evaporation at 37 °C in the presence of salt [45]. This approach avoids extreme temperatures or pH levels, which often lead to irreversible protein denaturation, nor cross-linking agents. It enables simple and versatile fabrication of materials with tunable properties for TE. In the present study, we aimed to develop a bilayered albumin-based scaffold composed of a porous layer seeded with patient-derived chondrocytes and a smooth layer. This bilayered design was conceived to reflect the structural organization of otorhinolaryngological tissues, combining a porous layer supporting cartilage-like formation with a dense smooth layer that may provide a supportive interface for epithelial coverage. We notably demonstrated that both epithelial or fibroblast cultures could be cultured on a smooth layer *in vitro* [45]. Here, bovine serum albumin (BSA) was used here as a model protein to demonstrate the feasibility of the salt-assisted fabrication approach. After characterization and biocompatibility assessment, patient-derived chondrocytes were seeded to evaluate their capacity to support cartilage-like formation *in vitro* and *in vivo* using a mouse model. While these results provide a proof-of-concept, further work will be required to validate this strategy using human-derived albumin under xeno-free and clinically relevant conditions. Our approach may contribute to ongoing efforts in cartilage tissue engineering and translational research.

## 2 Results and discussion

### 2.1 Albumin-based scaffolds are tunable porous scaffolds for tissue engineering

In this study, we developed a bilayered albumin-based scaffold using a salt-assisted compaction process, which is an efficient and cost-effective formulation method [45]. In this process, calcium chloride (CaCl_2_) is added to a bovine serum albumin (BSA) solution to generate materials that are water-insoluble, easily hydratable, and easy to handle. We previously demonstrated that various salts (*e.g.*, CaCl_2_, NaBr, KCl, MgCl_2_) can be used to adjust scaffold properties, including manageability, water uptake, and mechanical properties [44]. Although the biomaterial fabrication process is designed to eliminate all salts, CaCl_2_ was selected as a precautionary measure, as the next most suitable alternative involved the use of bromide during processing. The process does not require chemical or physical cross-linking agents or procedures and can be applied to other natural protein sources. Albumin-based materials can be molded into various forms, such as membranes, cuboids, cylinders, or tubes, and exhibit different structure types, including dense, smooth, or porous. To date, the mechanism underlying the formation of the albumin materials has not been fully elucidated; however, our hypotheses and results suggest that it is based on a process of salt-assisted protein compaction. Salt ions screen the charge patches present on the surface of albumin, thereby reducing electrostatic repulsion between protein molecules. This decrease in repulsive forces promotes protein–protein interactions and molecular proximity, leading to the compaction of the protein network. Structural analyses indicate that the secondary structures of albumin are preserved, suggesting that the process does not rely on protein denaturation but rather on a reorganization of intermolecular interactions. In addition, the appearance of inter-protein β-sheets suggests the formation of new structural interactions contributing to the cohesion and stabilization of the resulting network. Overall, salt-assisted compaction likely results from a balance between ion-induced electrostatic screening and the establishment of stabilizing protein–protein interactions, enabling the transition from soluble albumin to a compact, stable, and water-insoluble protein material.

**Fig. 1:**
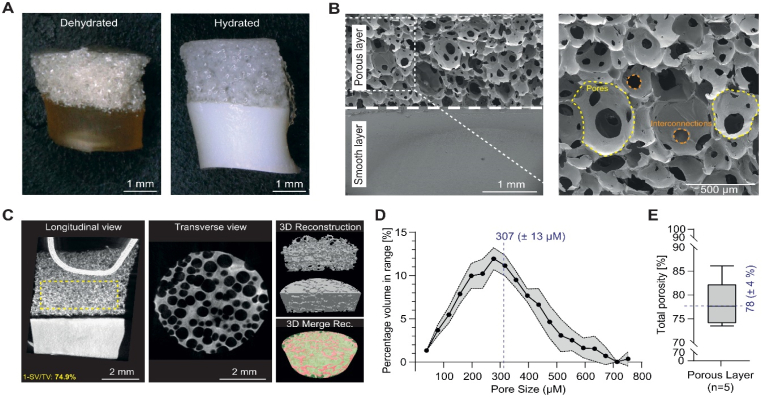
Macroscopic and microscopic views of the bilayered albumin-based scaffold. (A) Representative macroscopic images of the scaffold in both dehydrated and hydrated states. (B) Cross-sectional scanning electron microscopy (SEM) image illustrating the bilayered architecture, composed of a dense (smooth) upper layer and a porous lower layer. A higher magnification SEM image of the porous region highlights the internal pore morphology. Individual pores are delineated with yellow dashed circles and interconnected pore structures are indicated with orange dashed circles. Representative macroscopic and SEM images were selected from at least three independent experiments. (C) Longitudinal and transverse cross-sectional views with three-dimensional reconstructions obtained from micro-computed tomography (μCT) imaging. The longitudinal section shows the metallic syringe needle used for sample positioning during μCT acquisition, passing through the upper third of the sample. Representative μCT images were selected from five independent experiments. (D) μCT-derived pore size distribution and (E) porosity quantification of the porous layer. (n = 5).

We produced cylindrical albumin-based scaffolds consisting of two joined layers: a smooth layer and a porous layer (Fig. 1A). On the one hand, the smooth layer was intentionally included in the bilayered design because it plays several essential functional roles during the preparation and use of the scaffold. First, it maintains the geometry and structural integrity of the construct during *in vivo* maturation. Chondrocytes exert strong contractile forces that can cause fully porous albumin foams to collapse into small spherical aggregates, so here the smooth layer prevents this shrinkage and preserves the predefined cylindrical shape. Second, the smooth layer acts as a non-porous barrier that prevents cell loss during seeding by confining chondrocytes within the porous compartment, thereby ensuring efficient and homogeneous colonization of the scaffold. Third, it provides a rigid and stable surface that greatly facilitates handling and implantation, while protecting the more fragile porous layer during surgical manipulation. Together, these features make the smooth layer a temporary but critical structural component of the bilayered scaffold, even though it is designed to biodegrade after implantation. On the other hand, the porous layer represents the functional compartment of the scaffold, specifically designed to support chondrocyte proliferation and cartilage matrix deposition. Its architecture provides a large surface area and efficient pathways for nutrient and oxygen diffusion, which are essential for maintaining cell viability within the bulk of the construct. The spherical and open pore network facilitates uniform cell infiltration and promotes homogeneous ECM formation throughout the scaffold.

Briefly, a CaCl_2_/BSA mixture was prepared in a sodium acetate buffer. Part of it was poured into a mold to form the smooth layer. The remaining portion was emulsified to create a foam and poured directly onto the smooth layer to form the porous layer. The resulting bilayered preparation was then placed in an oven at 37 °C until complete water evaporation. The porous layer had a sponge-like structure (Fig. 1B and 1C). Scanning electron microscopy (SEM) and microcomputed tomography (μCT, Fig. 1C) images revealed the spherical geometry and interconnectivity of the pores, with visible holes (Fig. 1B and 1C). The resulting interconnected pores form a crucial accessibility network within the scaffold, facilitating deeper cell seeding and proliferation within the biomaterial and providing a large available surface area [46,47]. The formation of pores and interconnected pores is a direct consequence of the emulsification process, through the trapping of gas air bubbles. All pores had a similar spherical geometry but varied in size, with a distribution ranging from 40 to 760 μm and a mean diameter of 307 ± 13 μm (median diameter of 280 μm) (Fig. 1D). These values were obtained from μCT imaging and subsequent quantitative analysis using the CTAn software. The effect of pore size on cartilage regeneration has been studied, but the search for the optimal pore size remains inconclusive, as studies present contradictory findings. Although there is no consensus on the optimal pore size for cartilage TE, the 50 to 500 μm range is considered acceptable [48]. However, the impact of pore size on biological processes, such as proliferation, colonization, neocartilage formation, and differentiation, is multifactorial because it can also be influenced by the type of scaffold material used, its architecture, or the cell type.

Another key parameter to consider when designing 3D scaffolds is porosity [49]: the higher the porosity, the better the delivery of nutrients and oxygen, and consequently, the cell growth. Although previous studies have demonstrated that a porosity of 50% is sufficient for cell attachment, migration, and proliferation on scaffolds, a higher porosity, closer to 90%, is considered more favorable for cartilage repair [18,50,51]. The porosity of the complete albumin bilayered scaffold was first assessed using liquid displacement in absolute ethanol, a method ensuring no scaffold shrinkage or swelling. The bilayered scaffold exhibited a mean porosity of 61.8 ± 3.1% (data not shown). The calculation was then refined for each layer using μCT, revealing a porosity of 78.1 ± 4.4% for the porous layer (Fig. 1E), while the smooth layer exhibited no detectable porosity.

Efficient water absorption is also crucial for cartilage tissue-engineered scaffolds because it reflects the effectiveness of body fluid absorption and nutrient transport. We evaluated this property in our bilayered scaffolds through water immersion and monitoring for up to 48 h. The gross appearance of a dry (t = 0h) and fully hydrated scaffold (t = 48h or 2.880 min) are shown in Fig. 1A. In the dry state, the bilayered scaffold presented a characteristic translucent and slightly brownish smooth layer. After water immersion, a change in appearance was observed, characterized by a swelling of the two layers and a whitish opacification of the smooth layer (Fig. 1A, right). The measured water uptake at equilibrium was 257 ± 17% (Fig. S1, dots) and occurred in two stages. During the first stage (first 10 min of immersion), rapid water absorption was observed in the porous layer. During the second stage, water absorption took place over several hours in the smooth layer (Figure S1A). The best mathematical fit of our observations was achieved using a two-exponential model (Figure S1A; water uptake ≈250%), suggesting two concomitant yet kinetically distinct water uptake processes. The first was attributed to pore filling and foam hydration of the porous layer (Figure S1A “porous layer” and **S1.B** “porous layer kinetic” *t*_1_ ~ 12 min; water uptake ≈89%). The second was attributed to the hydration of the smooth layer (Figure S1A “smooth layer” and **S1.B** “smooth layer kinetic” *t*_2_ ~ 314 min; water uptake ≈161%). The bilayered scaffold reached equilibrium regarding water absorption within 24 h of immersion, as no significant additional increase was detectable at 48 h. Although the water absorbed by the scaffold could be released by mechanical force (*e.g.,* pressure applied with fingers), re-immersion in water restored its original shape and size, thereby exhibiting a shape-memory property (data not shown). This feature could be important for industrial processes and clinical uses that might require drying prior to sterilization.

### *2.2* *In vitro* cell viability, cell spreading, and proliferation occur throughout the albumin-based scaffold

**Fig. 2:**
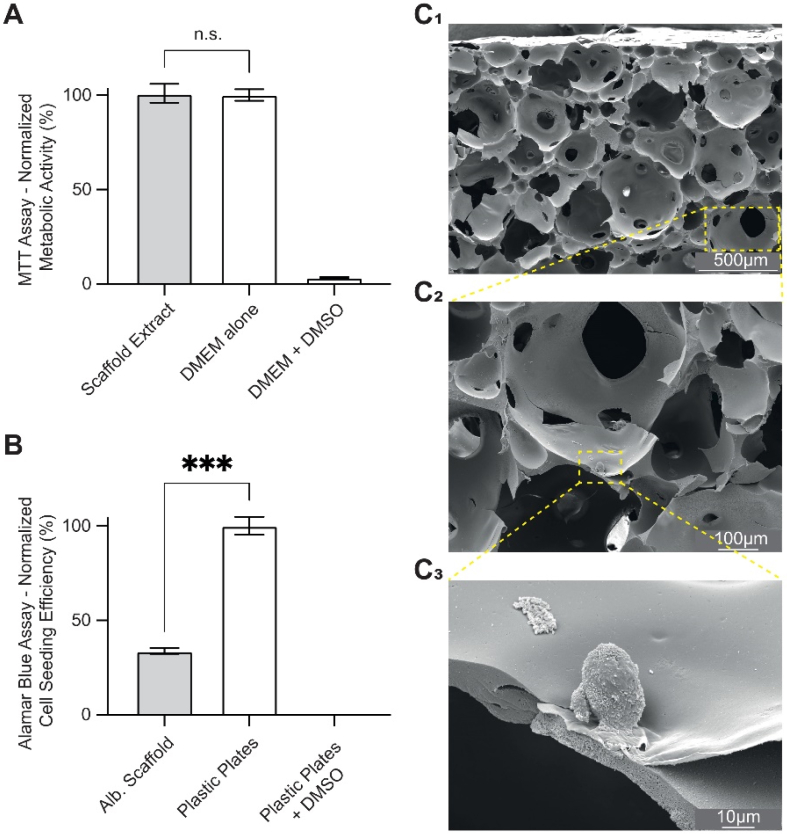
**Viability, adhesion, and proliferation of BALB/c-3T3 cells on the bilayered albumin-based scaffold.** (A) Normalized metabolic activity of BALB/c-3T3 cells after 24 h in contact with extracts from the scaffold. Metabolic activity was quantified using an MTT assay (3-[4,5-dimethylthiazol-2-yl]-2,5 diphenyl tetrazolium bromide). The absorbance obtained was normalized with a positive control (Ctrl+). In the positive control group, the cells were cultured for only 24 h in a cell culture medium. In the negative control (Ctrl-) group, the cells were cultured for only 24 h in a cell culture medium containing 20% dimethyl sulfoxide (*n*= 3). (B) Cell seeding efficiency of BALB/c-3T3 cells after 4 h of adhesion time on the scaffold. The fluorescence was quantified by an Alamar Blue assay. The obtained fluorescence was normalized with a positive control (Ctrl+). In the positive control group, the cells adhered to plastic cell culture plates for 4 h (*n* = 3). (C1 to C3) Cross-sectional scanning electron microscopy images of the porous layer of the base scaffold seeded with BALB/c 3T3 cells after 14 days of culture. DMEM: Dulbecco's Modified Eagle Medium.

We then investigated the potential of a bilayered albumin-based scaffold for TE applications. To assess its biocompatibility *in vitro*, we first determined whether the scaffold generated cytotoxic degradation products over time. The scaffold was incubated in a cell culture medium for 3 days. Then, the medium (referred to as “scaffold extract”) was applied to fibroblastic cells (BALB/c-3T3) for 24 h. Cellular metabolic activity was evaluated using the 3- [4,5-dimethylthiazol-2-yl]-2,5 diphenyl tetrazolium bromide (MTT) assay. No significant difference was observed compared to control conditions (Fig. 2A), suggesting that the scaffold does not release cytotoxic degradation compounds over a 3-day *in vitro* period, in accordance with the ISO 10993-5 guidelines.

As albumin is known to exhibit limited cell adhesion properties [17], we evaluated cell adhesion and proliferation on the scaffold. Fibroblastic cell (BALB/c-3T3) adhesion was evaluated by measuring cell seeding efficiency after 4 h of incubation and compared to a control. The seeding efficiency after 4 h was relatively low (29.3 ± 3.9%)(Fig. 2B) [52], and cells maintained a spherical morphology without spreading, as observed by SEM in Fig. 2C**_1-3_.** To improve initial cell attachment, an α-poly-L-lysine (α-PLL) coating was applied. This coating increases the density of positively charged groups at the scaffold surface, thereby promoting electrostatic interactions with negatively charged cell membranes [53]. Although albumin contains multiple lysine residues that are positively charged at neutral pH, most of these groups are not freely accessible for cell interaction [54]. Moreover, native albumin exhibits an overall negative net charge at physiological pH due to its high content of acidic amino acids. Consequently, the density and spatial distribution of available positive charges on the albumin surface are insufficient to promote strong electrostatic interactions with negatively charged cell membranes. To overcome this limitation, an exogenous α-PLL coating was applied to the albumin scaffold, forming a layer enriched in exposed amine groups. This treatment markedly increases the surface density of positive charges and provides a favorable interface for cell adhesion and spreading through electrostatic attraction.

**Fig. 3:**
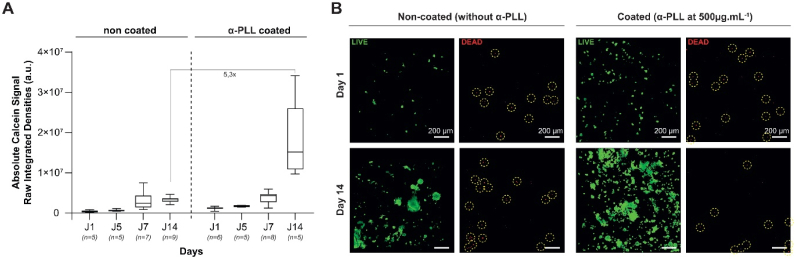
**Cell viability assessment within the porous layer of bilayered albumin-based scaffolds using LIVE/DEAD staining.** (A) Quantitative analysis of cell viability based on the raw integrated density of the calcein signal (green channel), representing metabolically active (live) BALB/c-3T3 cells. Data are presented as box plots, showing values from five to nine randomly selected regions per condition, across time points from day 1 (J1) to day 14 (J14), for scaffolds with and without α-poly-L-lysine (α-PLL) surface coating. (B) Representative confocal Z-stack projection images of the porous scaffold layer on days 1 and 14. Viable cells are stained with calcein (green), while non-viable cells are labeled with ethidium bromide (red). Ethidium-positive spots are highlighted with yellow circles for visualization purposes. (n represents the number of quantified fields obtained from two independent experiments for each condition)

The scaffold was incubated in an α-PLL solution (500 μg mL^−1^, 50 mM Tris, and 150 mM NaCl) for 1 h and rinsed prior to use. Cell viability, with and without α-PLL coating, was assessed using calcein/ethidium bromide staining over 14 days (Fig. 3). Both absolute or normalized signals were used as an approximation of the cell’s live/dead status of the cells, as full 3D segmentation was not feasible due to the formation of compact cell clusters within the scaffold. The use of MTT for direct scaffold analysis was avoided due to known interactions between albumin and tetrazolium salts, which can lead to false-positive results [55]. BALB/c-3T3 cells were directly cultured on top of the scaffolds for 14 days and imaged across multiple representative regions (1,400x1,400 μm) at a depth of 300 μm (Fig. 3B and S2A-B). Cells were distributed as numerous small and evenly dispersed patches, resulting in an overall homogeneous coverage across the scaffold. This is consistent with the previously demonstrated low cell affinity for albumin (Fig. 3A, day 1 and **S2.A,** day 5). The α-PLL coating slightly increased both calcein and ethidium bromide signals, maintaining an equivalent live/dead normalized ratio during the first week (Figure S2C). This can be attributed to the more spread-out form of the cells in the coating, visible as early as day 1 (Fig. 3A, S2.A). Cell clusters were visible, particularly in the non-coated condition (Figures S2A, day 7 and **3.B**, day 14). Reconstructed 3D images revealed that fibroblastic cells could colonize across at least the first 300 μm of the porous scaffold within a week, (Figure S2B, face and 90° left turn rotation on day 7). This demonstrates that the porous layer of the albumin-based scaffold supports cell infiltration, which is in line with the presence of a dense network of interconnected pores. Dead cells were observed at all times (Fig. 3B and S2A). The ethidium bromide signal from the non-coated scaffold on day 1 was used as the reference value for normalization and indicated comparable live/dead ratios between coated and non-coated scaffolds during the first week (Figure S2C). Thus, the scaffolds exhibited high long-term viability under both conditions. We confirmed the previously observed non-cytotoxicity of the non-coated scaffold and demonstrated that adding an α-PLL surface coating does not compromise its biocompatibility. Although no significant differences in the absolute or relative calcein signals were observed during the first week, the effectiveness of the α-PLL coating on fibroblast proliferation was evident by day 14, with a 5.3-fold increase in raw integrated density (Fig. 3A). Consistently, normalized live/dead signal ratios were around 3.1 in the non-coated condition, increasing to 16.9 in the α-PLL-coated condition (Figure S2C). Based on these observations, we concluded that an α-PLL coating enhances both cell adhesion, as evidenced by a more spread-out fibroblast phenotype, and cell proliferation when compared with the uncoated scaffold. Therefore, the bilayered albumin-based scaffold was coated with α-PLL for the rest of the study.

### 2.3 Human nasal chondrocytes show proliferation and undergo chondrogenic redifferentiation *In vitro* on bilayered albumin-based scaffolds

We aimed to develop a strategy to support cartilage regeneration, with a focus on cartilage hyaline-like formation. To this end, we seeded human nasal chondrocytes (HNCs) on the porous scaffold layer. Then, we cultured the HNCs in an enriched “proliferation” medium for 1 week to promote cell growth. The medium was enriched with fibroblast growth factor (FGF)-2 and transforming growth factor (TGF)-β1, as previously described [38]. FGF-2 not only enhances chondrocyte proliferation but also modulates actin stress fibers, thereby facilitating the redifferentiation of dedifferentiated chondrocytes toward a hyaline phenotype [56]. Meanwhile, TGF-β1 plays a crucial role in driving chondrocyte differentiation and ECM synthesis, including type II collagen and proteoglycans, while regulating hypertrophic maturation to maintain stable cartilage tissue [57]. Finally, the combined use of FGF-2 and TGF-β should promote rapid chondrocyte expansion while preserving their differentiation potential and cartilage-specific matrix production [58].

**Fig. 4:**
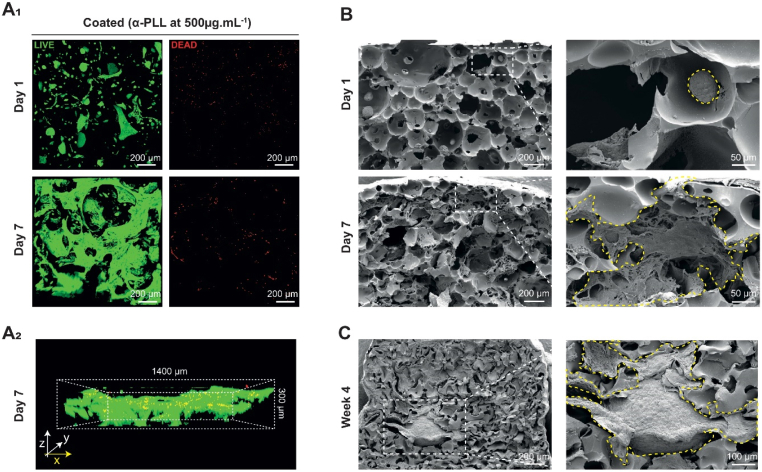
**Viability, adhesion, and proliferation of human nasal chondrocytes (HNCs) on the bilayered albumin-based scaffold**. (A_1_) Z-stack confocal images of the scaffold after LIVE/DEAD staining, showing cell viability of HNCs. (A_2_) 3D images on day 7, viewed from the lateral side. Living cells are stained in green and dead cells are highlighted in red. (B) Cross-sectional scanning electron microscopy (SEM) images of the scaffold’s porous layer on days 1 and 7, with magnification. HNCs are represented by yellow dashed circles. C. Cross-sectional SEM images at week 4. α-PLL: α-poly-L-lysine.

Cell viability was estimated as described above, using calcein (green) to label living cells and ethidium bromide (red) to identify dead cells. These results are presented in Fig. 4. On day 1, the porous scaffold layer presented high cell viability but a scattered HNC distribution (Fig. 4A_1_, day 1). By day 7, we observed a higher number of viable cells displaying a more spread morphology (Fig. 4A_1_, day 7) throughout the entire 300 μm evaluated (Fig. 4A_2_), suggesting sustained cell viability. These results indicate that the scaffold supports HNC viability on both days 1 and 7, which may be related to the enriched medium conditions. Although dead cells are present at both time points, this was consistent with previous observations in fibroblasts and did not prevent colonization.

In addition, SEM images reveal the microscopic morphology and distribution of cells within the scaffold. On day 1, we observed low cell colonization — consistent with the limited seeding efficiency (Fig. 2B). HNCs were spherical and clustered (Fig. 4B, day 1 + zoom) and were unevenly distributed but distributed but observed across the entire thickness of the porous layer (Fig. S3). This observation suggests rapid cell infiltration into the entire porous network post-seeding due to capillary forces and sedimentation. However, as no direct tracking of cell trajectories was performed, active cell migration within the scaffold cannot be excluded, particularly during early tissue organization processes. By day 7, we observed an increase in cell number (Fig. 4B, day 7), with HNCs more evenly distributed, adherent and spread-out on the scaffold’s porous layer (Fig. 4B, day 7 - zoom). Overall, these observations indicate that the scaffold is compatible with cell survival and colonization. This may be beneficial for initiating *in vitro* chondrogenic redifferentiation, as cell density is considered an important parameter for cartilage formation. High cell density (the higher, the better) and viability are essential to improve the efficiency of chondrogenesis and cartilage production [59,60].

**Fig. 5:**
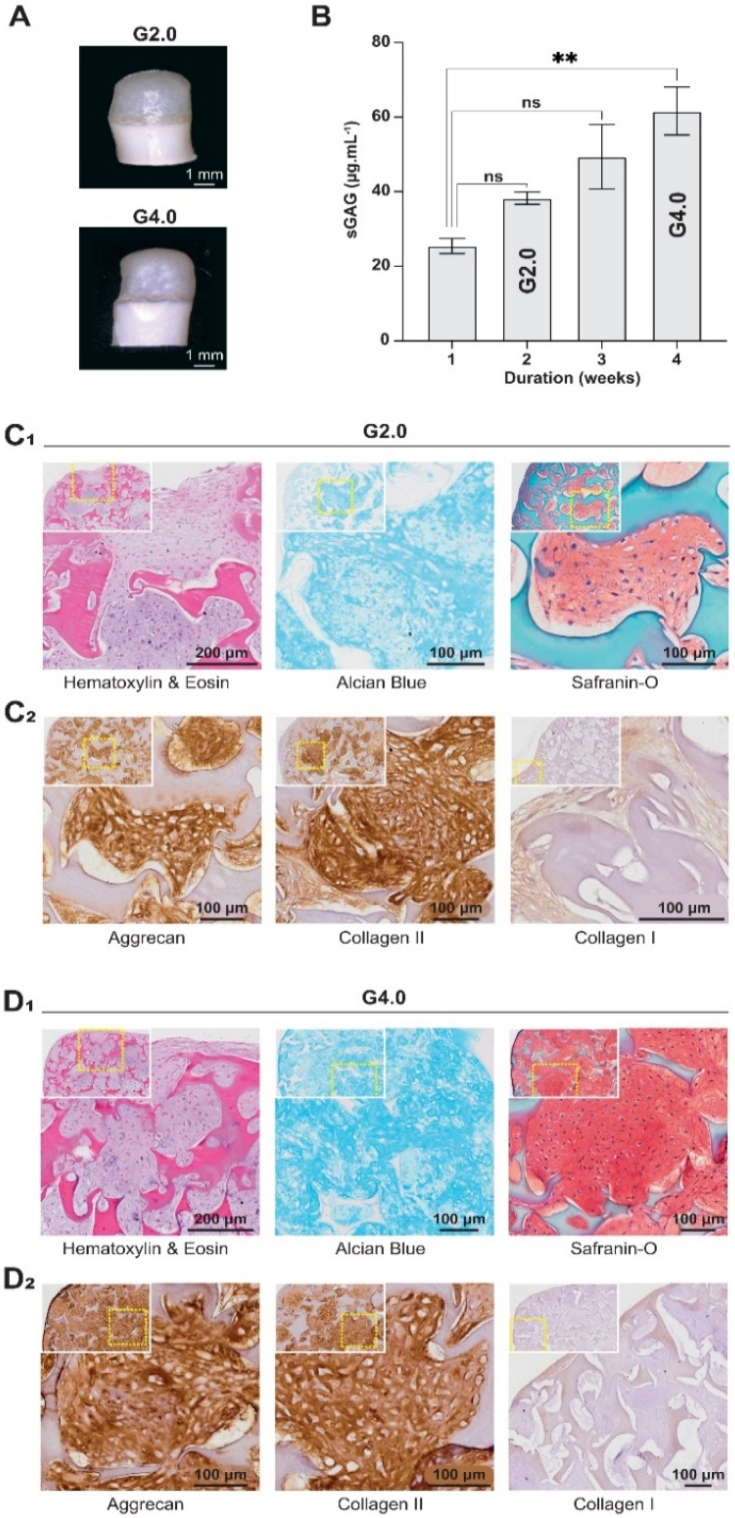
***In vitro* evaluation of the porous layer of the bilayered albumin-based scaffolds after 2 (G2.0) and 4 weeks (G4.0) of chondrogenic redifferentiation.** (A) Macroscopic images of *in vitro* cartilage-like tissues formed on the porous scaffold layer after 2 and 4 weeks of *in vitro* chondrogenic redifferentiation. (B) Quantification of sulfated glycosaminoglycans **(**sGAGs) in the tissue-engineered construct at 1, 2, 3, and 4 weeks of *in vitro* chondrogenic redifferentiation and performed by using the 1,9-dimethylmethylene blue assay (*n*= 3). ∗∗*p* < 0.01, *t*-test. (C_1_ and D_1_) Hematoxylin/eosin (H&E), Alcian Blue (AB), and Safranin-O (SO) stainings were used to evaluate sGAG deposits. (C_2_ and D_2_) Immunohistochemical stainings for aggrecan, collagen type II, and collagen type I. Positive staining appears brown (diaminobenzidine tetrahydrochloride). Each histological image includes an inset showing a zoomed-out overview of the sample (about 7 × for G2.0 and 5.5 × for G4.0).

Next, we investigated the albumin-based scaffold’s ability to promote *in vitro* chondrogenic redifferentiation and cartilage regeneration. As described above, an initial 1-week proliferation step was performed to promote HNC proliferation and ensure good scaffold colonization prior to *in vitro* chondrogenic redifferentiation. After this 1-week proliferation step, the HNCs were cultured in an enriched “differentiation” medium for 4 weeks. The redifferentiation medium contained ascorbic acid, dexamethasone, and TGF-β3 to enhance chondrogenic differentiation. Ascorbic acid and dexamethasone stimulate the synthesis of cartilaginous matrix, while TGF-β3 added to the medium inhibits chondrocyte terminal differentiation and hypertrophy [61,62]. In the following part, the scaffolds will be named “GX.Y", with “X" representing *in vitro* culture time and “Y" representing *in vivo* culture time. Macroscopic observations of the porous layer of G2.0 and G4.0 samples appear smoother, more opaque, and pearly-white (Fig. 5A), features commonly associated with cartilage-like tissue**.** The pores were progressively filled with HNCs that produced new ECM (Fig. 4C). Hematoxylin/Eosin (HE) histological analysis showed homogeneous cell colonization at both G2.0 and G4.0 (Fig. 5C1 and 5D1). HNCs displayed a predominantly round morphology, although some cells with a more immature phenotype were still observed at G2.0. These immature chondrocytes displayed a more elongated or polygonal shape, smaller and less defined lacunae, and a more homogeneous pericellular matrix [63]. Alcian blue (AB) and Safranin-O (SO) stainings were used to assess the sGAG content of the tissue-engineered construct. They confirmed the presence of sGAGs, with more intense and homogeneous staining at G4.0, suggesting increased ECM deposition over time (Fig. 5C1 and 5D1). This was further supported by quantitative sGAG measurements, which increased from 25.1 ± 2.1 to 61.1 ± 6.4 μg sGAG.mL^−1^ between 1 week and 4 weeks in *in vitro* chondrogenic redifferentiation (Fig. 5B). Immunohistochemical analysis showed stronger expression of cartilage-associated markers (collagen type II and aggrecan) at G4.0 compared to G2.0 (Fig. 5C2 and 5D2), consistent with progressive matrix maturation. In contrast, collagen type I expression remained low and mainly localized int the peripheral zone (PZ), with no significant signal in the core zone (CZ), suggesting limited fibrocartilage-like features under these conditions (Fig. 5C2 and 5D2).

### 2.4 Albumin-based scaffolds support cell viability and are biocompatible with host tissue

**Fig. 6:**
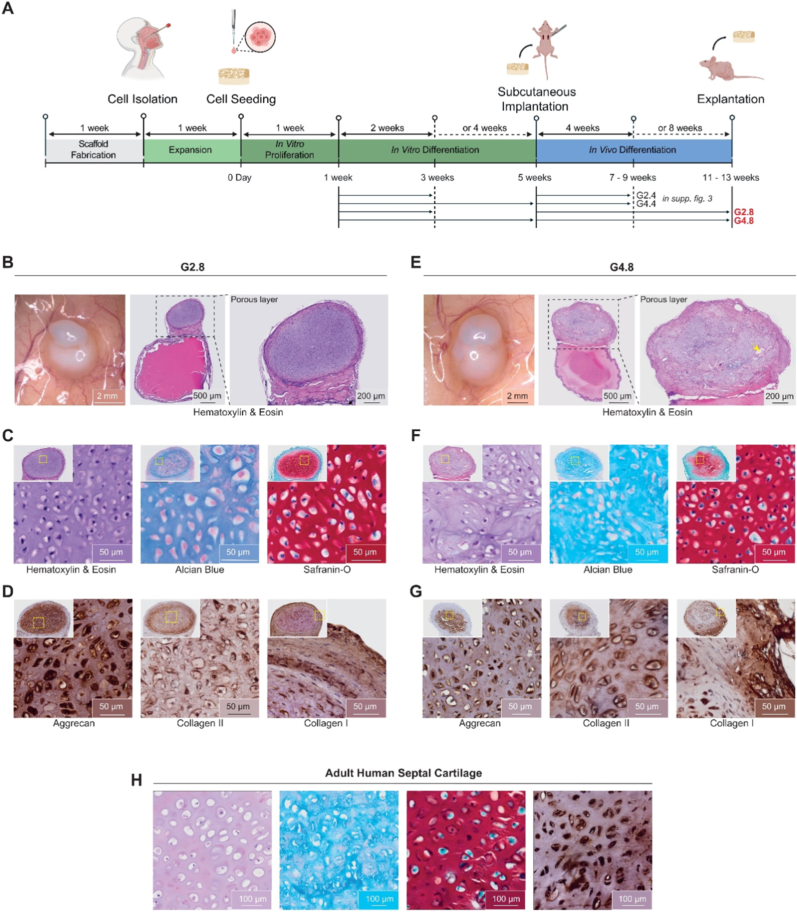
***In vivo* evaluation of the porous layer of the albumin-based scaffold after 2 or 4 weeks of *in vitro* chondrogenic redifferentiation and 8 weeks of subcutaneous implantation in nude mice (G2.8, n = 7 mice and G4.8, n = 7 mice).** (A) Schematic overview of the experimental design. Created with BioRender.com and finalized in Adobe Illustrator. (B and E) Macroscopic images of the cartilage-like tissue that formed on the porous scaffold layer after 8 weeks of implantation. (C and F) Histological analysis of explants using hematoxylin/eosin (H&E), Alcian Blue (AB), and Safranin-O (SO) stainings to evaluate tissue morphology and glycosaminoglycan content. (D and G) Immunohistochemical stainings for the cartilage-specific aggrecan, collagen type II, and collagen type I markers. Positive immunoreactivity is visualized by brown diaminobenzidine tetrahydrochloride chromogen. Each histological image includes an inset showing a zoomed-out overview of the sample (about 15.5 × for both G2.8 and G4.8). (H) Histological and immunohistochemical stainings (H&E, AB, SO, and aggrecan) of adult human septal cartilage serving as a positive reference control.

We conducted investigations in nude mice by subcutaneously implanting the tissue-engineered constructs to assess biocompatibility, functionality, cartilage development and scaffold degradation. As described above, HNCs were seeded on the porous layer of the bilayered albumin-based scaffolds, proliferated during 1 week, and were then cultured for 2 (G2.Y) or 4 weeks (G4.Y) *in vitro*. The tissue-engineered constructs were implanted subcutaneously in the dorsum of athymic nude mice (*n* = 5), with up to two scaffolds per mouse. They were harvested 4 (G2.4 and G4.4) or 8 weeks (G2.8 and G4.8) after implantation. Additional mice (*n* = 5) received scaffolds without cells to serve as control mice (G0.4 and G0.8). The study protocol is presented in Fig. 6A.

Qualitative histological observations suggested a progressive degradation of the albumin scaffold over time. In acellular scaffolds, the porous layer was no longer detectable after 4 weeks *in vivo* (G0.4), while the smooth layer remained present. By 8 weeks (G0.8), the smooth layer appeared progressively eroded, and by 12 weeks (G0.12), no residual material was observed, suggesting near-complete scaffold degradation within approximately 2 to 3 months (Fig. S4). Differences in degradation behavior between the two layers may be related to variations in albumin density. A similar trend was observed in cellularized scaffolds. The smooth layer remained visible at early time points (G2.4), became progressively eroded at 8 weeks (G2.8, G4.8), and was no longer detectable at 12 weeks (G2.12). In parallel, the porous layer appeared progressively populated by HNCs as early as 4 weeks and appeared progressively replaced by newly formed tissue, which exhibited features consistent with cartilage-like ECM over time. Long-term observations at 6 months (G2.24) showed features consistent with hyaline-like cartilage in the apparent absence of residual scaffold. These observations suggest that scaffold degradation occurs concomitantly with the development of cartilage-like tissue, with no marked differences observed between acellular and cell-seeded scaffolds at the evaluated time points. This degradation process is essential for chondrocytes to rebuild healthy and functional cartilage. In cartilage TE, scaffold degradation is generally expected to be coordinated with extracellular matrix deposition. While degradation over 3–6 months is often recommended [64], the shorter window observed here (2–3 months) may reflect the ectopic subcutaneous implantation model, which does not replicate the full mechanical and biological environment of a cartilage defect.

Gross tissue examination suggested the presence of blood vessels on the surface on the scaffold surface and indicated integration within the subcutaneous tissue (Fig. 6B and 6E). In all conditions, we observed the formation of a thin fibrous capsule containing fibroblasts and macrophages around the outer part of the scaffold (Fig. S5 – Gomori & CD68). The recruitment and proliferation of host fibroblasts led to the encapsulation of the biomaterial within a fibrous matrix, the thickness of which has been associated with the intensity of the inflammatory response and to the physicochemical properties of the implanted material [65,66]. According to the literature, the presence of a thin and well-organized capsule is generally considered indicative of a controlled host response, reflecting a typical wound-healing integration process [67]. In this context, our observations are consistent with a moderate host response and initial tissue compatibility. This moderate response was expected and plays a role in the healing process.

Immunohistochemical staining revealed a relatively low presence of CD68^+^ macrophages around the implant, with a decrease in density between weeks 4 and 8, suggesting a reduction in inflammatory activity over time (Fig. S5). Additionally, a network of newly formed blood vessels was observed on the scaffold’s surface. However, CD31 staining showed limited vascularization within the implanted construct, with only sparse vascular structures present inside the scaffold (Fig. S5 – CD31**)**. This observation is consistent with the intrinsically low vascularity of cartilage tissue and may indicate that the neoformed tissue retains cartilage-like characteristics. The limited vascularization may also contribute to maintaining a cartilage phenotype, as excessive vascularization is often associated with undesired tissue remodeling such as ossification - which is a common challenge in cartilage regeneration efforts [68]. We acknowledge that the use of a nude mouse model limits the assessment of the full immune response. It limits the interpretation of immune compatibility and prevents a comprehensive evaluation of immunological responses. Therefore, the present results should be interpreted as a preliminary assessment of host response, and further studies in immunocompetent models will be required to fully address biocompatibility and translational potential.

### 2.5 Albumin-based scaffolds enhance cartilage regeneration In vivo from HNCs

The aim of the subcutaneous implantation study was also to investigate the cartilage-forming capacity and phenotype maintenance of HNCs within the scaffold. Immune-deficient mice have been used for various applications in cartilage research [69]. After 8 weeks of implantation, the tissue-engineered construct (TEC) macroscopically resembled cartilage-like tissue (Fig. 6B and 6F). For G2.8 and G4.8 conditions, histological analyses revealed the presence of lacuna-like structures in HE staining, together with cartilage-associated extracellular matrix deposition, as indicated by AB and SO staining (Fig. 6C and 6F), and expression of collagen II and aggrecan (Fig. 6D and 6G). These features were qualitatively comparable to those observed in human septal cartilage samples (Fig. 6H), suggesting a certain level of tissue maturation. In contrast, samples implanted for 4 weeks (Figure S6 - G2.4 and G4.4) showed lower levels of matrix deposition and marker expression, consistent with a less advanced maturation state (Fig. S6). These results suggest that *in vivo* implantation contributes significantly to cartilage maturation, rather than being solely dependent on the duration of *in vitro* culture.

To further assess tissue quality, the Bern Histological Score (0–9 scale) was used [[70], [71], [72]] (Fig. S7–E). Total scores ranged from 0 (fibrous, noncartilaginous tissue) to 9 (good hyaline cartilage tissue). The Bern score enables finer discrimination of subtle tissue quality variations compared with other systems (*e.g.*, O’Driscoll [73]), as it covers a broader scoring range (3–9 vs. 0–3) [70]. G2.8 samples reached 7.17 ± 0.92, while G4.8 samples reached 6.43 ± 0.81, with no significant difference between groups (Fig. S7–A). Although progressive maturation was observed, long-term evaluation remains necessary to assess tissue stability and potential phenotypic changes. These results suggest that extending *in vitro* redifferentiation from 2 to 4 weeks did not result in a measurable improvement in final tissue quality after implantation. Instead, cartilage maturation appears to be more strongly influenced by implantation time *in vivo*, supporting the concept that the body functions as an optimal bioreactor supporting the concept that the *in vivo* environment may act as a favorable bioreactor. Previous studies report optimal culture periods of 1–3 weeks, and prolonged *in vitro* maturation has been suggested to potentially affect *in vivo* outcomes despite improved *in vitro* results [74]. This finding is particularly relevant for TE applications, as it means that culture phases can be shortened without compromising the quality of the cartilage obtained. In oncology, where time is a critical factor, this represents a major advantage regarding the acceleration of tissue reconstruction.

Although progressive maturation was observed, long-term evaluation remains necessary to assess tissue stability and potential phenotypic changes. Indeed, long-term excessive vascularization could promote hypertrophy and calcification [75]. To further explore long-term outcomes, additional constructs were implanted for 6 months (G2.24). Histological analysis showed a homogeneous cartilage-like tissue without signs of vascular invasion or necrosis, suggesting maintenance of tissue integrity over time. The Bern score reached 8.75 ± 0.19, suggesting a high level of maturation closely resembling native hyaline cartilage, approaching the maximum Bern Score (Fig. S7). However, these observations remain primarily descriptive and do not allow a comprehensive assessment of long-term phenotypic stability. As such, theses findings be interpreted as indicative of cartilage-like tissue rather than a confirmed hyaline cartilage phenotype, and a fibrocartilaginous component cannot be excluded.

In addition, no orthotopic defect model was evaluated in this study. The subcutaneous implantation model was intentionally used as a first *in vivo* step to assess biocompatibility, stability, and cartilage-like tissue formation in a controlled environment [74,76]. This model is particularly relevant for non-load-bearing applications such as nasal or auricular cartilage reconstruction [77,78]. Future studies will be required to evaluate the performance of the scaffold in clinically relevant defect models and under mechanical loading conditions. In this context, a two-step strategy—ectopic pre-maturation followed by orthotopic transplantation—is increasingly recognized as an effective approach to ensure tissue maturation and functional integration before load-bearing implantation [79]. Therefore, our study represents a necessary and relevant preliminary step before advancing to defect-specific and long-term *in vivo* evaluations.

In conclusion, our tissue-engineered constructs were organized into two distinct showing features comparable to native cartilage architecture (Fig. S8) [80]: (i) cells in the PZ and (ii) cells on the CZ. Each zone exhibited specific differences in cell morphology, organization, and ECM deposition.

(i) In the PZ of our tissue-engineered construct, cells were small, elongated, and arranged parallel to the surface. This organization is reminiscent of perichondrium-like structures, which are characterized by fibroblast-like cells and collagen type I-rich ECM. Immunohistochemical analysis confirmed collagen type I expression in this region, supporting this interpretation. In native cartilage, the perichondrium consists of an outer fibrous layer (previously described) and an inner chondrogenic layer containing progenitor cells capable of differentiating into chondrocytes (except in fibrous cartilage) [81,82]. The perichondrium plays an important role in cartilage nutrition and vascular supply, although it is often absent in tissue-engineered constructs [83]. Few previous studies have addressed the formation of perichondrium-like regions in neocartilage constructs. Some authors obtained similar results to ours and succeeded in developing scaffold-free cartilaginous constructs with a perichondrium-like region [52].
(ii) In the CZ of our tissue-engineered, cells exhibited a markedly different morphology, as this region was densely populated with cells presenting a single, central, larger, and oval-shaped nucleus. Cells were distributed within an abundant basophilic extracellular matrix and appeared separated from each other, consistent with a cartilage-like organization. Cells were observed within lacunae-like structures, although these features may be influenced by fixation-related artifacts [[84], [85], [86]]. Overall, this region showed characteristics compatible with chondrocyte-like morphology and matrix deposition. They were surrounded by an increasingly basophilic, homogeneous, and amorphous material (appearing more purple) gradually separating them. Notably, the cells were embedded in an abundant basophilic matrix and located individually in lacunae matrix cavities. Lacunae were the result of shrinkage artifacts during the fixation. The cell membrane was actually in direct contact with the peripheral membrane.

## 3 Conclusion

In this study, we developed and evaluated a new bilayered albumin-based scaffold for cartilage tissue engineering in otorhinolaryngology, using salt-assisted compaction process. This scaffold combined a smooth layer, previously shown to support epithelial culture *in vitro* [45], and facilitating practical handling, and a porous layer provided an interconnected 3D environment for chondrocyte infiltration, colonization, viability, and matrix deposition. The scaffold supported cell viability, with no cytotoxic effects. *In vitro*, HNCs cultured exhibited features of hyaline cartilage-like matrix production, including type II collagen and aggrecan. *In vivo*, subcutaneous implantation showed a moderate inflammatory response, and progressive tissue maturation over time. The TEC exhibited two distinct zones: the PZ and the CZ, an organization reminiscent of native cartilage structure. Overall, these results support the feasibility of this bilayered scaffold as a platform for cartilage TE. However, these findings should be interpreted as indicative of cartilage-like tissue rather than a confirmed hyaline cartilage phenotype, and a fibrocartilaginous component cannot be excluded.

While these results support the feasibility of the proposed approach, several limitations should be considered. Due to the thickness of the regenerated tissue, mechanical testing could not be reliably performed. In addition, the use of BSA as a model protein and a nude mouse model may restrict the assessment of immune responses and long-term biocompatibility. Importantly, for clinical translation, the scaffold is intended to combine patient-derived HNCs with GMP-grade human albumin. These findings should therefore be considered as a proof-of-concept, and further studies in clinically relevant conditions are required. In conclusion, we have developed a bilayered albumin-based scaffold as a proof-of-concept platform for cartilage tissue engineering. While further validation is required, this approach provides a basis for future developments in otorhinolaryngological applications, including nasal, auricular, and tracheal reconstruction.

## 4 Materials and methods

### 4.1 Fabrication of bilayered albumin-based scaffolds

**Fig. 7:**
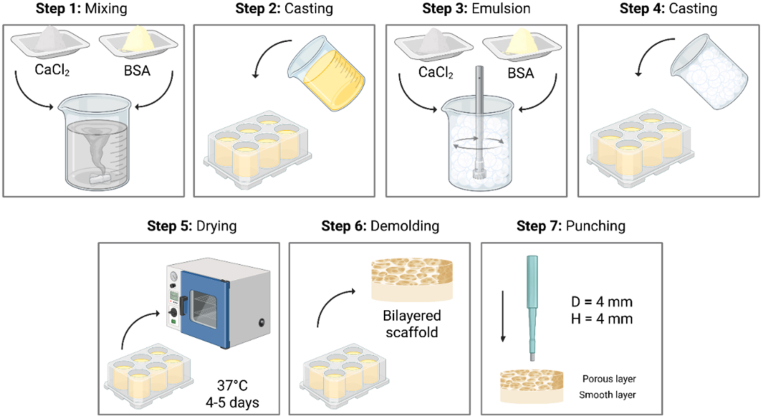
**Seven-step fabrication protocol of bilayered albumin-based membranes.** Created with BioRender.com. BSA: bovine serum albumin.

A solution with BSA (PM-T1726, Biosera, France) at a concentration of 200 mg mL^−1^ and calcium chloride (CaCl_2_, 2 H_2_O) (223506, Sigma-Aldrich, France) at a concentration of 2 mol L^−1^ ([CaCl_2_/BSA] molar ratio = 700) was prepared separately in a sodium acetate buffer (pH = 6.00, 20 mmol L^−1^).Then, equal volumes of the two solutions were mixed by adding the CaCl_2_ solution dropwise to the BSA solution while stirring to prevent salt precipitation. The formulation of the bilayered albumin-based scaffold was as follows (Fig. 7).

1) Formulation of the smooth layer: the CaCl_2_/BSA mixture was poured into a mold at a precise grammage of 113 mg cm^−2^;
2) Formulation of the porous layer: immediately after step 1, the same CaCl_2_/BSA mixture was emulsified. The foam formed was placed directly onto the mixture from step 1. The assembly was placed in an oven at 37 °C until complete water evaporation (4-5 days).

Washing (with distilled water) was necessary to remove all the salt, leaving a water-insoluble albumin-based scaffold. After washing, scaffolds were punched into their final cylindrical, bilayered form using a 5 mm biopsy punch. They were then sterilized under sterile conditions using MilliQ water and 70% ethanol and stored in sterile Milli-Q water at 4 °C.

### 4.2 Physico-chemical characterization of bilayered albumin-based scaffolds

#### 4.2.1 Morphological characterization

Cross-sectional morphology, pore size, and interconnectivity of the bilayered albumin-based scaffold were observed using a scanning electron microscope (QuantaTM 250 FEG, FEI, USA). Pore size was determined by using ImageJ to measure pore diameter on three samples, based on SEM images of a cross-section. A Kruskal-Wallis test (non-parametric one-way ANOVA) indicated no statistically significant difference (*p* = 0.403) among these three samples. Therefore, measurements (*n* = 311) were pooled to determine the mean pore size and standard deviation (SD).

#### 4.2.2 Water absorption

The scaffolds (*n* = 4) were cut into discs (4 mm in diameter and 4-6 mm thick). Briefly, the samples were dried at 37 °C to a constant dry mass (*M*_dry_). The dry samples were then placed in a 24-well plate and immersed in the same amount of distilled water (2 mL). The samples were removed at different immersion times (*t* = 10, 30, 60, 360 min, 24, and 48 h) and blotted on paper to remove excess water. For each immersion time, samples were weighed and the hydrated mass was recorded. The water uptake was defined by:

(1) $$W\left(t\right)\left(t\right)=\frac{M\left(t\right)‐M\left(0\right)}{M\left(0\right)}\times 100$$

where *M*(*t*) represents the hydrated sample mass at time *t* and *M*(0) represents the dry mass. The means and SDs of four independent experiments are represented as solid dots and error bars in Fig. S1. In these experiments, the average maximum water absorption was 257 ± 17%, representing almost 2.6 ± 0.2 times the dry mass of the scaffolds.

The experimental uptake kinetics suggested an exponential rise to a maximum. However, as we observed that the two parts of the scaffold did not swell at the same rate, we modeled the measurements using a four-parameter expression involving two exponentials:

(2) $$W\left(t\right)=a\left[1-\exp\left(\frac{t}{t_{1}}\right)\right]+b\left[1-\exp\left(\frac{t}{t_{2}}\right)\right]$$

where *a* represents the contribution of the porous layer to the total absorption *W*, *t*_1_ represents the characteristic absorption time, *b* represents the contribution of the smooth layer to the total absorption *W,* and *t*_2_ corresponds to the characteristic absorption time.

#### 4.2.3 μCT-derived porosity and pore size distribution

Contrast-enhanced microcomputed tomography (CEμCT) is an advanced non-invasive imaging technique that provides high-resolution 3D images, analysis, and visualization of biological samples, such as cartilaginous tissue. This technique involves using X-rays, that travel through the sample and are subsequently detected to generate a 2D digital projection. Using contrast-enhancing agents is common to address the issue of low X-ray attenuation in soft tissues, due to their intrinsic low density [87]. In this study, phosphotungstic acid (PTA) was used to enhance the contrast of the μCT images. PTA can penetrate constructs and bind to proteins, resulting in contrast enhancement between the empty pores and the surrounding protein structure, thus facilitating visualization and analysis [[88], [89], [90]]. Using PTA as a contrast-enhancing agent offers two key advantages over other compounds. Firstly, it is non-toxic, and secondly, it is compatible with H&E staining. This property enables histological analysis following μCT scans without removing the PTA staining [91,92]. However, PTA staining can also result in tissue shrinkage and affect the sample's mechanical properties. PTA functions in both 100% aqueous solutions and ethanol. However, the additional dehydration caused by ethanol use should be avoided to minimize tissue shrinkage [93].

All samples were prepared using a 6 mm punch, followed by PTA staining to enhance their X-ray attenuation. Firstly, a fresh solution of 5% w/v PTA in ultrapure water was prepared before each staining. The PTA solution was stored at ambient temperature and used within 7 days. Prior to scanning, each sample was immersed in 50 mL of PTA solution and left without agitation for 24 h. The sample was then subjected to a 1-min washing procedure involving deionized water. Subsequently, the sample was stored in a 1.5-mL cryotube containing deionized water. To prevent sample movement during μCT scanning, samples were secured in the cryotubes by inserting a metallic syringe needle through the porous sample segment (see Fig. 1C top left, white dense element).

μCT imaging was conducted using a Skyscan 1172 μCT system (Skyscan N.V., Aartselaar, Belgium, SkyScan1172 Micro-CT control software). Cryotubes were stabilized with Blu-Tack putty (Bostik, Smart Adhesives) on the sample holder within the Skyscan 1172. The 9.9x6-μm resolution and pixel size were carefully selected to achieve a balance between the desired detail level and the overall image dimensions. A 0.5-mm Al filter was applied to remove noise from low-energy photons and enhance image quality. We selected the appropriate voltage and current settings with the objective of maximizing contrast while minimizing the presence of noise artifacts in the resulting projection images. The voltage and current of the X-ray source were set to 59 kV and 167 μA, respectively. During scanning, random vertical movement and averaging options were enabled to minimize artifacts and improve the signal-to-noise ratio. New flat-field values for an empty image acquisition frame were acquired and used for flat-field correction. These steps ensured high-quality 180° μCT imaging for subsequent analysis and interpretation.

The NRecon image reconstruction program (Skyscan N.V., Aartselaar, Belgium, Version 1.6.5.7) facilitated the reconstruction of the μCT scans and cross-section images were generated from the tomographic projection scans. Settings for scan reconstruction were carefully selected: a smoothing factor of 3 to reduce noise, an attenuation of 0-0.08, a ring artifact reduction of 5 to compensate for varying X-ray attenuation due to different material densities, a beam hardening correction of 0%, a smoothing filter of 3, and misalignment compensation to account for any misalignments in the acquired projections. The desired reconstruction area was specified to ensure that the reconstruction encompassed the upper and lower slices of interest. The file format for the reconstructed image was set to 8-bit BMP. For morphological analysis and visualization, the reconstructed files were transferred to the CTAn software (Skyscan N.V., Aartselaar, Belgium, V1.11.10.0+). To calculate foam porosity, we selected a region of interest for each sample, ensuring that it was entirely within the foam's structure. Using a grey value threshold was instrumental in binarizing the positive and negative regions, thereby segmenting the pores and allowing the subsequent calculation of the overall porosity.

To calculate the pore size distribution, the following procedure was carried out in CTAn [Bruker, Bruker microCT Method Note: Porosity analysis, 2014]. The threshold was inverted so that the pores were represented as a solid structure, which was then analyzed. The “trabecular thickness” tool within CTAn software was then used to determine the pores’ “local thickness distribution” [94].

### 4.3 Biological characterization of bilayered albumin-based scaffolds

#### 4.3.1 Cell cytotoxicity

According to the ISO 10993-5 standard, a metabolic activity higher than 70% indicates that a material is not cytotoxic. The scaffolds were cut into discs (4 mm in diameter and 4-6 mm thick), sterilized by successive washes with sterile Milli-Q water and 70% (v/v) ethanol, and then stored in sterile Milli-Q water at 4 °C until use. The scaffolds were dried at room temperature and weighed on the day of use. They were then transferred to a 24-well plate, immersed in culture medium, and incubated for 72 h at 37 °C, with 5% CO_2_, and 95% relative humidity. According to the ISO10993-12 standard, the samples were immersed in a specific volume at a rate of 1 mL of culture medium per 0.1 g of dry sample to assess the cytotoxicity of the extracts. The term “extract” refers to the culture medium in which the samples were incubated. If the samples can absorb water, the culture medium volume should be increased by the volume that the sample can absorb. After incubation, the “extract” was brought into contact with fibroblasts (BALB/c-3T3), cultured in a 96-well plate at a density of 6000 cells/well for 24 h at 37 °C. Positive and negative controls were prepared using complete culture medium and culture medium containing an additional 20% dimethyl sulfoxide (DMSO) (v/v). The culture medium was then removed from each well and replaced with 100 μL of MTT solution (1:10 dilution) for 2 h at 37 °C. The formazan crystals produced were solubilized in 100 μL DMSO for 15 min at room temperature with stirring. Absorbance was measured at 570 nm using a plate reader (VLBL00GD2, Varioskan, Thermo Fisher Scientific, France). The quantity of formazan produced, and thus the absorbance measured, is directly proportional to the cell’s metabolic activity and, therefore, to the number of living cells.

#### 4.3.2 Cell viability

The discs were prepared as described above and then coated by immersion in an α-PLL solution (500 μg/mL in 50 mM Tris, 150 mM NaCl) for 1 h. They were subsequently rinsed twice with the same buffer solution and used directly without further processing. On the day of use, the scaffolds were transferred to a 24-well plate with a hydrophobic poly(hema) coating to prevent cell adhesion to the wells’ bottom. The scaffolds were blotted on paper to remove excess water. Cell viability and distribution on scaffolds were assessed using the viability/cytotoxicity kit (L3224, Invitrogen, Thermo Fisher Scientific, France) according to the manufacturer’s protocol. The scaffolds were seeded with fibroblasts (BALB/c 3T3) at a density of 10,000 cells/scaffold. Then, the scaffolds were placed in an incubator at 37 °C for 45 min to let the cells adhere. After that, the growth medium (Dulbecco's modified Eagle's medium (DMEM) high glucose + 10% fetal bovine serum (v/v) + 1% (v/v)) was loaded in each well and renewed every 2 days. The medium was removed for analysis on days 1, 7, and 14. Cell-seeded scaffolds were incubated with 4 mM calcein AM (staining living cells in green) and 2 mM ethidium homodimer (staining dead cells in red) in 6 mL of phosphate buffered saline (PBS) for 20-25 min at room temperature. After that, the scaffolds were placed in the sagittal plane, and Z-stack images with an average depth of 200-300 μm were acquired using a confocal microscope (LSM 510, Zeiss, Germany). A total of 20 Z-stack images, taken at 5.5 μm intervals, were obtained from each scaffold. Two Z-stack acquisitions were taken for each of the duplicated scaffolds. LIVE/DEAD viability analysis was conducted using full-stack projections (five to nine regions per experimental condition), generated via the Zeiss ZEN imaging software. Fluorescence images were separated into green (calcein) and red (ethidium bromide) channels and homogeneously thresholded to minimize background signal arising from the slight autofluorescence of the albumin-based scaffold. Thresholding was applied by excluding the lowest 20% of signal intensity in the green channel and the lowest 30% in the red channel. Image quantification was then performed using ImageJ (version 1.54p). Raw integrated density values were extracted for each channel and used as either absolute values or values normalized relative to day 1 measurements.

### 4.4 *In vitro* evaluation of cartilage regeneration

#### 4.4.1 Isolation of human nasal chondrocytes

HNCs were isolated from nasal septum biopsies obtained from patients undergoing rhinoplasty. Briefly, the cartilage biopsies were minced and the chondrocytes were isolated using 0.15% (w/v) type II collagenase (10 mL g^−1^ of tissue) for 22 h and resuspended in DMEM containing 5% (v/v) human platelet lysate, 2 U mL^−1^ heparin, 4.5 mg mL^−1^ D-Glucose, 0.1 mM nonessential amino acids, 1 mM sodium pyruvate, 10 mM HEPES buffer, 100 U mL^−1^ penicillin, 100 μg mL^−1^ streptomycin, and 0.29 mg mL^−1^ L-glutamine (complete medium). The isolated chondrocytes were counted using trypan blue, plated in tissue culture flasks at a density of 10^4^ cells.cm^−2^ and cultured in complete medium, with the addition of 1 ng mL^−1^ of TGF-β1, 5 ng mL^−1^ of FGF-2 in a humidified incubator at 37 °C, with 5% CO_2_ and 95% relative humidity.

#### 4.4.2 Cell seeding and culture of human nasal chondrocytes on bilayered albumin-based scaffolds

The scaffolds were cut into discs (4 mm in diameter and 4-6 mm thick), sterilized by successive washes using sterile water and 70% (v/v) ethanol, and stored at 4 °C. Then, the scaffolds were transferred to a 24-well plate with a hydrophobic poly(HEMA) coating to prevent cell adhesion. HNCs (passage 2 or 3) were directly seeded onto the porous scaffold layer following thawing for culture and redifferentiation. For *in vitro* experiments, cells from four independent donors were used across the different assays. This approach was adopted to avoid donor-dependent effects and to ensure that the observed outcomes were reproducible across multiple biological sources. Donor selection was also dependent on cell availability from established stocks. The HNCs were left for 1 week in contact with a proliferation medium: DMEM (10938025, Gibco) with 4.5 mg mL^−1^ glucose content supplemented with 10% (v/v) fetal bovine serum, 1% (v/v) penicillin-streptomycin-glutamine (10378016, Gibco), 10 mM HEPES (156630080, Gibco), 1 mM sodium pyruvate (11360039, Gibco), and growth factors such TGF-β1 (240-B-010/CF, Biotechne, USA) at 1 ng mL^−1^ of and FGF-2 (233-FB-010/CF, Biotechne) at 5 ng mL^−1^. They were then left during 2 or 4 weeks in contact with a differentiation medium: DMEM (10938025, Gibco) with 4.5 mg mL^−1^ glucose content supplemented with 1% (v/v) ITS+1 (I2521, Sigma-Aldrich), 1% (v/v) penicillin-streptomycin-glutamine (10378016, Gibco), 10 mM HEPES (156630080, Gibco), 1 mM (11360039, Gibco), and growth factors such ascorbic acid (A8960, Sigma-Aldrich) at 100 μM, dexamethasone (D4902, Sigma-Aldrich) at 100 nM, and TGF-β3 (100-36E, Peprotech, France) at 10 ng mL^−1^.

HNCs were harvested and suspended in a culture medium to obtain a concentration of 500,000 cells per 15 μL droplet. HNCs were then seeded at a density of 500,000 cells/scaffold onto the porous scaffold surface. The scaffolds were placed in an incubator for 2 h at 37 °C, with 5% CO_2_ and 95% relative humidity. Meanwhile, the sample absorbed the droplet containing the cells. After incubation, 1.5 mL of proliferation medium was added to each well and renewed twice weekly. At least two scaffolds seeded with HNCs were cultured for each analysis time point. After each time point, the scaffolds were removed from the incubator and sent for experimentation.

#### 4.4.3 Gross appearance

At the end of the culture period, digital microscope photographs of the various samples were taken before they were fixed for histological and immunohistochemical evaluations.

#### 4.4.4 Histological and immunohistochemistry

The scaffolds were fixed in 4% (v/v) paraformaldehyde for 24 h at room temperature for histological evaluations. They were then dehydrated in ethanol baths with increasing concentrations (water, 70% ethanol, 95% ethanol, and 100% ethanol) before being immersed in toluene and embedded in paraffin (39602004, Leica Biosystems, France). Samples were cross-sectioned to a thickness of 5 μm using a microtome (RM2125 RTS, Leica). These sections were recovered and spread on SuperFrost Plus slides (Fisher Scientific, 10149870). The corresponding sections were stained with H&E to assess tissue structure, AB (0.1% (w/v), pH = 1), and SO (0.1 % (w/v)) to locate the sGAG deposits, and Gomori’s trichrome (0.3% (w/v)) to detect collagen type I and identify fibrotic areas.

For immunohistochemical evaluations, the protocol was similar to that described above. Collagen I was revealed using a mouse monoclonal anti-human type I collagen antibody (dilution 1:200 in PBS, sc-293182, Santa Cruz Biotechnology, USA), collagen II using a mouse monoclonal anti-human type II collagen antibody (dilution 1:100 in PBS, sc-52658, Santa Cruz Biotechnology), and aggrecan using a mouse monoclonal anti-human aggrecan antibody (dilution 1:200 in PBS, sc-33695, Santa Cruz Biotechnology, United States). A horseradish peroxidase (HRP)-conjugated anti-mouse antibody was then applied as a secondary antibody. The ABC complex (avidin/biotin complex, PK-7200, Vestastain Laboratories) was added and left for 30 min. Diaminobenzidine tetrahydrochloride (DAB) (SK-4105, Vector Laboratories) was used as the chromogen for color development, followed by counterstaining with H&E. The stained samples were finally observed under an optical microscope (Olympus BX60).

Ki-67 was detected using a rabbit anti-human Ki-67 monoclonal antibody (1:100 in PBS, RM-9106-SO, Epredia, Thermo Fisher Scientific). An HRP-conjugated anti-rabbit antibody (1:200 in PBS, Santa Cruz Biotechnology) was then applied as a secondary antibody. The ABC complex was added and left for 1 h. DAB was used as the chromogen for color development. Finally, the stained samples were observed under an optical microscope (Olympus BX60).

#### 4.4.5 Sulfated glycosaminoglycan assay

Following the manufacturer's protocol, the sGAG content was determined using the Blyscan™ dye-binding assay (Biocolor Ltd., Carrickfergus, UK). sGAGs were extracted from the samples using a papain reagent diluted in 0.2 M sodium phosphate buffer (Na_2_HPO_4_- NaH_2_PO_4_, pH = 6.4). Each scaffold was incubated with 1 mL of papain extraction solution (0.1 mg mL^−1^), then placed in a water bath at 65 °C for 18 h. After centrifugation at 10,000 g for 10 min, 50 μL of the supernatant was added to 1 mL of the Blyscan dye reagent and then shaken for 30 min at room temperature. An additional centrifugation (13,000 g, 15 min) was necessary to sediment the bluish precipitate formed. The supernatant was discarded, and 500 μL of dissociation reagent was added. After complete dissolution and centrifugation (13,000 g, 10 min), 200 μL of each sample were transferred to a clear-bottomed 96-well plate in duplicate. Absorbance was measured with a plate reader at 656 nm. The sGAG content (expressed in μg.mL^−1^) was calculated using a standard curve established with each plate reading.

### *4.5* *In vivo* evaluation of cartilage regeneration

The cartilage formation capacity of the bilayered albumin-based scaffold *in vivo* was assessed using a subcutaneous implantation assay in nude mice (APAFIS #46421). HNCs were seeded on the porous layer of the bilayered albumin-based scaffolds and cultivated for 2 (G2.X) or 4 weeks (G4.X) *in vitro*. For *in vivo* experiments, cells from four additional independent donors were used across the different assays. This approach was adopted to avoid donor-dependent effects and to ensure that the observed outcomes were reproducible across multiple biological sources. Donor selection was also dependent on cell availability from established stocks. The scaffolds were implanted subcutaneously in the dorsum of athymic nude mice, with up to two scaffolds per mouse (9-week-old females). Different conditions were investigated during the study: G0.4 (n = ), G0.8 (n = ), G2.4 (n = 12), G2.8 (n = 7), G4.4 (n = 12), G4.8 (n = 7), and G2.24 (n = 7). The mice were anesthetized with isoflurane and a subcutaneous buprenorphine injection. Two incisions were made in the skin lateral to the dorsal midline at the level of the hip joint after analgesia with lidocaine (2 mg mL^−1^) and bupivacaine (2 mg mL^−1^). The constructs were placed subcutaneously, and the skin was sutured with absorbable thread. Buprenorphine (0.1 mg kg^−1^) was administered via subcutaneous injection after surgery. After 4 or 8 weeks, the animals were euthanized under gaseous anesthesia with isoflurane and injection of a lethal Euthasol dose (140 mg kg^−1^). The explants were then collected and fixed for 2 h in 4% paraformaldehyde before being embedded in paraffin and subjected to histological and immunohistological analyses as previously described.

### 4.6 Semi-quantification analysis

Histological quality was assessed using the modified Bern score (0–9 scale), which evaluates cartilage matrix intensity and uniformity (SO), extracellular matrix organization, and cell morphology (Table in Figure S7E). To obtain a representative and spatially unbiased evaluation of each sample, each histological section was subdivided into square regions of interest (ROIs) measuring 250 μm × 250 μm. Each ROI was independently scored according to the Bern criteria, yielding a local score ranging from 0 to 9.

For each sample, the Bern score was calculated as the mean of all ROIs within the section, providing a global histological assessment of the entire construct. Scoring was performed independently and in a blinded manner by two trained evaluators, and the final Bern score corresponded to the average of both assessments. For each condition (GX.Y), the reported Bern score represents the mean value obtained from three to four independent samples (denoted as “N” in Fig. S7). Statistical analyses were performed using two-way ANOVA followed by Tukey’s multiple-comparisons test.

### 4.7 Statistical analysis

All experiments were conducted in triplicate unless otherwise specified. Data analysis was performed using GraphPad Prism (GraphPad Software, USA). The results are presented as mean ± SD or standard error of the mean, as indicated in the figure legends. Statistical comparisons were performed either using *t*-test or one-way analysis of variance (ANOVA), followed by the appropriate post hoc test when applicable. Statistical significance was defined as follows: ∗p < 0.05, ∗∗p < 0.01, ∗∗∗p < 0.001, and ∗∗∗∗p < 0.0001.

## CRediT authorship contribution statement

**Christelle Bertsch:** Conceptualization, Data curation, Formal analysis, Investigation, Methodology, Validation, Visualization, Writing – original draft, Writing – review & editing. **Florent Colin:** Data curation, Formal analysis, Methodology, Project administration, Visualization, Writing – original draft, Writing – review & editing. **Eya Aloui:** Methodology, Resources. **Julien Graff:** Investigation, Methodology. **Maria Cristina Antal:** Conceptualization, Investigation, Methodology, Validation, Writing – review & editing. **Sabine Kuchler-Bopp:** Data curation, Methodology, Visualization, Writing – review & editing. **Adrien Moya:** Data curation, Methodology, Resources, Writing – review & editing. **Romy Marek:** Conceptualization, Data curation, Formal analysis, Methodology, Validation, Visualization, Writing – original draft. **Sven Zaugg:** Data curation, Formal analysis, Investigation, Visualization. **Eric Mathieu:** Investigation, Visualization. **Claire Thibault:** Investigation. **Christian Debry:** Conceptualization, Supervision. **Jordan Beurton:** Methodology, Resources, Writing – review & editing. **Bernard Senger:** Formal analysis, Investigation, Validation, Visualization, Writing – review & editing. **Benoit Frisch:** Writing – review & editing. **Michael de Wild:** Funding acquisition, Investigation, Methodology, Validation, Writing – original draft, Writing – review & editing. **Arnaud Scherberich:** Conceptualization, Data curation, Funding acquisition, Methodology, Resources, Supervision, Writing – original draft, Writing – review & editing. **Philippe Lavalle:** Conceptualization, Funding acquisition, Project administration, Supervision, Validation, Writing – original draft, Writing – review & editing. **Léa Fath:** Conceptualization, Formal analysis, Funding acquisition, Project administration, Supervision, Validation, Writing – original draft, Writing – review & editing.

## Declaration of competing interest

The authors declare the following financial interests/personal relationships which may be considered as potential competing interests: Lavalle reports financial support from Interreg. Fath reports financial support from the French National Research Agency (ANR), the Carnot Institute MICA, ITI HiFunMat, and Cancéropôle EST. All other authors declare that they have no known competing financial interests or personal relationships that could have influenced the work reported in this paper.

## Data Availability

Data will be made available on request.

## References

[1] Dehghani F., Fathi A., Li Q., Mai Y.-W. (2017). Biomaterials for Implants and Scaffolds.

[2] Kang S.-W., Bada L.P., Kang C.-S., Lee J.-S., Kim C.-H., Park J.-H., Kim B.-S. (2008). Articular cartilage regeneration with microfracture and hyaluronic acid. Biotechnol. Lett..

[3] Batty L., Dance S., Bajaj S., Cole B.J. (2011). Autologous chondrocyte implantation: an overview of technique and outcomes. ANZ J. Surg..

[4] Krill M., Early N., Everhart J.S., Flanigan D.C. (2018). Autologous Chondrocyte implantation (ACI) for knee cartilage defects: a review of indications, technique, and outcomes. JBJS Rev..

[5] Glenn R.E., McCarty E.C., Potter H.G., Juliao S.F., Gordon J.D., Spindler K.P. (2006). Comparison of fresh osteochondral autografts and allografts: a canine model. Am. J. Sports Med..

[6] Chung C., Burdick J.A. (2008). Engineering cartilage tissue. Adv. Drug Deliv. Rev..

[7] Bertsch C., Maréchal H., Gribova V., Lévy B., Debry C., Lavalle P., Fath L. (2023). Biomimetic bilayered scaffolds for tissue engineering: from current design strategies to medical applications. Adv. Healthc. Mater..

[8] Hutmacher D.W. (2000). Scaffolds in tissue engineering bone and cartilage. Biomaterials.

[9] Wasyłeczko M., Sikorska W., Chwojnowski A. (2020). Review of synthetic and hybrid scaffolds in cartilage tissue engineering. Membranes.

[10] Yari D., Ebrahimzadeh M.H., Movaffagh J., Shahroodi A., Shirzad M., Qujeq D., Moradi A. (2022). Biochemical aspects of scaffolds for cartilage tissue engineering; from basic science to regenerative medicine. Arch. Bone Jt. Surg..

[11] Liu X., Zhang Z., Shi Y., Meng X., Qiu Z., Qu X., Dang J., Zhang Y., Sun L., Wang L., Zhu D., Mi Z., He J., Fan H. (2022). Effect of electrohydrodynamic printing scaffold with different spacing on chondrocyte dedifferentiation. Ann. Transl. Med..

[12] Benya P.D., Shaffer J.D. (1982). Dedifferentiated chondrocytes reexpress the differentiated collagen phenotype when cultured in agarose gels. Cell.

[13] Gagner J.E., Kim W., Chaikof E.L. (2014). Designing protein-based biomaterials for medical applications. Acta. Biomater.

[14] Wei F., Liu S., Chen M., Tian G., Zha K., Yang Z., Jiang S., Li M., Sui X., Chen Z., Guo Q. (2021). Host response to biomaterials for cartilage tissue engineering: key to remodeling. Front. Bioeng. Biotechnol..

[15] Kong F., Mehwish N., Lee B.H. (2023). Emerging albumin hydrogels as personalized biomaterials. Acta. Biomater..

[16] Evans T.W. (2002). Review article: albumin as a drug—biological effects of albumin unrelated to oncotic pressure. Aliment. Pharmacol. Ther..

[17] Kuten Pella O., Hornyák I., Horváthy D., Fodor E., Nehrer S., Lacza Z. (2022). Albumin as a biomaterial and therapeutic agent in regenerative medicine. Int. J. Mol..

[18] Zhang Q., Lu H., Kawazoe N., Chen G. (2014). Pore size effect of collagen scaffolds on cartilage regeneration. Acta Biomater..

[19] Chicatun F., Griffanti G., McKee M.D., Nazhat S.N., Ambrosio L. (2017). Biomedical Composites.

[20] Zhou Z., Cui J., Wu S., Geng Z., Su J. (2022). Silk fibroin-based biomaterials for cartilage/osteochondral repair. Theranostics.

[21] Montaseri Z., Abolmaali S.S., Tamaddon A.M., Farvadi F. (2023). Composite silk fibroin hydrogel scaffolds for cartilage tissue regeneration. J. Drug. Deliv. Technol..

[22] Cheng G., Davoudi Z., Xing X., Yu X., Cheng X., Li Z., Deng H., Wang Q. (2018). Advanced silk fibroin biomaterials for cartilage regeneration. ACS Biomater. Sci. Eng..

[23] Rojas-Murillo J.A., Simental-Mendía M.A., Moncada-Saucedo N.K., Delgado-Gonzalez P., Islas J.F., Roacho-Pérez J.A., Garza-Treviño E.N. (2022). Physical, mechanical, and biological properties of fibrin scaffolds for cartilage repair. Int. J. Mol. Sci..

[24] Barbon S., Stocco E., Macchi V., Contran M., Grandi F., Borean A., Parnigotto P.P., Porzionato A., De Caro R. (2019). Platelet-rich fibrin scaffolds for cartilage and Tendon regenerative medicine: from bench to bedside. Int. J. Mol. Sci..

[25] Roslan M.R., Nasir N.F.M., Cheng E.M., Amin N.A.M. (2016). 2016 International Conference on Electrical, Electronics, and Optimization Techniques (ICEEOT).

[26] Müller F.A., Müller L., Hofmann I., Greil P., Wenzel M.M., Staudenmaier R. (2006). Cellulose-based scaffold materials for cartilage tissue engineering. Biomaterials.

[27] Cordeiro R., Alvites R.D., Sousa A.C., Lopes B., Sousa P., Maurício A.C., Alves N., Moura C. (2023). Cellulose-based scaffolds: a comparative study for potential application in articular cartilage. Polymers.

[28] Iravani S., Varma R.S. (2022). Cellulose-based composites as scaffolds for tissue engineering: recent advances. Molecules.

[29] Kim Y., Zharkinbekov Z., Raziyeva K., Tabyldiyeva L., Berikova K., Zhumagul D., Temirkhanova K., Saparov A. (2023). Chitosan-based biomaterials for tissue regeneration. Pharmaceutics.

[30] Garcia Garcia C.E., Lardy B., Bossard F., Soltero Martínez F.A., Rinaudo M. (2021). Chitosan based biomaterials for cartilage tissue engineering: chondrocyte adhesion and proliferation. Food Hydrocoll. Health.

[31] Ressler A. (2022). Chitosan-based biomaterials for bone tissue engineering applications: a short review. Polymers.

[32] Wang M., Deng Z., Guo Y., Xu P. (2022). Designing functional hyaluronic acid-based hydrogels for cartilage tissue engineering. Mater. Today. Bio..

[33] Tsanaktsidou E., Kammona O., Kiparissides C. (2022). Recent developments in hyaluronic acid-based hydrogels for cartilage tissue engineering applications. Polymers.

[34] Li H., Qi Z., Zheng S., Chang Y., Kong W., Fu C., Yu Z., Yang X., Pan S. (2019). The application of hyaluronic acid-based hydrogels in bone and cartilage tissue engineering. Adv. Mater. Sci. Eng..

[35] Irawan V., Sung T.-C., Higuchi A., Ikoma T. (2018). Collagen scaffolds in cartilage tissue engineering and relevant approaches for future development. J. Tissue Eng. Regen. Med..

[36] Scotti C., Wirz D., Wolf F., Schaefer D.J., Bürgin V., Daniels A.U., Valderrabano V., Candrian C., Jakob M., Martin I., Barbero A. (2010). Engineering human cell-based, functionally integrated osteochondral grafts by biological bonding of engineered cartilage tissues to bony scaffolds. Biomaterials.

[37] Šećerović A., Pušić M., Kostešić P., Vučković M., Vukojević R., Škokić S., Sasi B., Vukasović Barišić A., Hudetz D., Vnuk D., Matičić D., Urlić I., Mumme M., Martin I., Ivković A. (2021). Nasal Chondrocyte–based engineered grafts for the repair of articular cartilage “Kissing” lesions: a pilot large-animal study. Am. J. Sports Med..

[38] Mumme M., Barbero A., Miot S., Wixmerten A., Feliciano S., Wolf F., Asnaghi A.M., Baumhoer D., Bieri O., Kretzschmar M., Pagenstert G., Haug M., Schaefer D.J., Martin I., Jakob M. (2016). Nasal chondrocyte-based engineered autologous cartilage tissue for repair of articular cartilage defects: an observational first-in-human trial. Lancet.

[39] Ashraf M.A., Shen B., Raza M.A., Yang Z., Amjad M.N., Din G.U., Yue L., Kousar A., Kanwal Q., Hu Y. (2025). Albumin: a review of market trends, purification methods, and biomedical innovations. CIMB.

[40] Sleep D., Cameron J., Evans L.R. (2013). Albumin as a versatile platform for drug half-life extension. Biochimica et Biophysica Acta (BBA) - General Subjects.

[41] Kratz F. (2008). Albumin as a drug carrier: design of prodrugs, drug conjugates and nanoparticles. J. Contr. Release.

[42] Fanali G., Di Masi A., Trezza V., Marino M., Fasano M., Ascenzi P. (2012). Human serum albumin: from bench to bedside. Mol. Aspect. Med..

[43] Prasopdee T., Sinthuvanich C., Chollakup R., Uttayarat P., Smitthipong W. (2021). The albumin/starch scaffold and its biocompatibility with living cells. Mater. Today Commun..

[44] Li P.-S., Lee I. -Liang, Yu W.-L., Sun J.-S., Jane W.-N., Shen H.-H. (2014). A novel albumin-based tissue scaffold for autogenic tissue engineering applications. Sci. Rep..

[45] Aloui E., Beurton J., Medemblik C., Hugoni L., Clarot I., Boudier A., Arntz Y., De Giorgi M., Combet J., Fleith G., Mathieu E., Kharouf N., Kocgozlu L., Heinrich B., Favier D., Brender M., Boulmedais F., Schaaf P., Frisch B., Lavalle P. (2025). Salt‐compact albumin as a new pure protein‐based biomaterials: from design to in vivo studies. Adv. Healthcare Mater..

[46] Turnbull G., Clarke J., Picard F., Riches P., Jia L., Han F., Li B., Shu W. (Will) (2017). 3D bioactive composite scaffolds for bone tissue engineering. Bioact. Mater..

[47] Zhang H.-C., Yu C.-N., Liang Y., Lin G.-X., Meng C. (2019). Foaming behavior and microcellular morphologies of incompatible SAN/CPE blends with supercritical carbon dioxide as a physical blowing agent. Polymers.

[48] Mukasheva F., Adilova L., Dyussenbinov A., Yernaimanova B., Abilev M., Akilbekova D. (2024). Optimizing scaffold pore size for tissue engineering: insights across various tissue types. Front. Bioeng. Biotechnol..

[49] Yadav P., Beniwal G., Saxena K.K. (2021). A review on pore and porosity in tissue engineering. Mater. Today Proc..

[50] Pan Z., Duan P., Liu X., Wang H., Cao L., He Y., Dong J., Ding J. (2015). Effect of porosities of bilayered porous scaffolds on spontaneous osteochondral repair in cartilage tissue engineering. Regen. Biomater..

[51] Oh S.H., Kim T.H., Im G.I., Lee J.H. (2010). Investigation of pore size effect on chondrogenic differentiation of adipose stem cells using a pore size gradient scaffold. Biomacromolecules.

[52] Ding C.-M., Zhou Y., Tan W.-S. (2005). Effects of seeding methods on seeding efficiency and initial cell distribution in 3-D scaffolds. Chin. J. Biotechnol..

[53] Lebaudy E., Guilbaud-Chéreau C., Frisch B., Vrana N.E., Lavalle P. (2023). The high potential of ε‐Poly‐ l ‐Lysine for the development of antimicrobial biomaterials. Adv. NanoBiomed. Res..

[54] Theodore Peters Jr. (1995). All About Albumin: Biochemistry, Genetics, and Medical Applications.

[55] Funk D., Schrenk H.-H., Frei E. (2007). Serum albumin leads to false-positive results in the XTT and the MTT assay. Biotechniques.

[56] Martin I., Suetterlin R., Baschong W., Heberer M., Vunjak‐Novakovic G., Freed L.E. (2001). Enhanced cartilage tissue engineering by sequential exposure of chondrocytes to FGF‐2 during 2D expansion and BMP‐2 during 3D cultivation. J of Cellular Biochemistry.

[57] Li T.-F., O'Keefe R.J., Chen D. (2009).

[58] Jakob M., Démarteau O., Schäfer D., Hintermann B., Dick W., Heberer M., Martin I. (2001). Specific growth factors during the expansion and redifferentiation of adult human articular chondrocytes enhance chondrogenesis and cartilaginous tissue formation in vitro. J. Cell. Biochem..

[59] Isaeva E.V., Beketov E.E., Yuzhakov V.V., Arguchinskaya N.V., Kisel A.A., Malakhov E.P., Lagoda T.S., Yakovleva N.D., Shegai P.V., Ivanov S.A., Kaprin A.D. (2021). The use of collagen with high concentration in cartilage tissue engineering by means of 3D-Bioprinting. Cell. Tiss. Biol..

[60] Okubo R., Asawa Y., Watanabe M., Nagata S., Nio M., Takato T., Hikita A., Hoshi K. (2019). Proliferation medium in three-dimensional culture of auricular chondrocytes promotes effective cartilage regeneration in vivo. Regen. Ther..

[61] Sulcanese L., Prencipe G., Canciello A., Cerveró-Varona A., Perugini M., Mauro A., Russo V., Barboni B. (2024). Stem-Cell-Driven chondrogenesis: perspectives on amnion-derived cells. Cells.

[62] Du X., Cai L., Xie J., Zhou X. (2023). The role of TGF-beta3 in cartilage development and osteoarthritis. Bone Res..

[63] Jessop Z.M., Zhang Y., Simoes I.N., Al-Sabah A., Badiei N., Gazze S.A., Francis L., Whitaker I.S. (2019). Morphological and biomechanical characterization of immature and mature nasoseptal cartilage. Sci. Rep..

[64] Welsh B.L., Sikder P. (2025). Advancements in cartilage tissue engineering: a focused review. J. Biomed. Mater. Res. B Appl. Biomater..

[65] Bota P.C.S., Collie A.M.B., Puolakkainen P., Vernon R.B., Sage E.H., Ratner B.D., Stayton P.S. (2010). Biomaterial topography alters healing *in vivo* and monocyte/macrophage activation *in vitro*. J Biomedical Materials Res.

[66] Ratner B.D. (2002). Reducing capsular thickness and enhancing angiogenesis around implant drug release systems. J. Contr. Release.

[67] Anderson J.M., Rodriguez A., Chang D.T. (2008). Foreign body reaction to biomaterials. Semin. Immunol..

[68] Pei Y.A., Chen S., Pei M. (2022). The essential anti-angiogenic strategies in cartilage engineering and osteoarthritic cartilage repair. Cell. Mol. Life Sci..

[69] Cavalli E., Fisch P., Formica F.A., Gareus R., Linder T., Applegate L.A., Zenobi-Wong M. (2018). A comparative study of cartilage engineered constructs in immunocompromised, humanized and immunocompetent mice. J. Immunol. Regen. Med..

[70] Rutgers M., van Pelt M.J.P., Dhert W.J.A., Creemers L.B., Saris D.B.F. (2010). Evaluation of histological scoring systems for tissue-engineered, repaired and osteoarthritic cartilage. Osteoarthr. Cartil..

[71] Power L., Acevedo L., Yamashita R., Rubin D., Martin I., Barbero A. (2021). Deep learning enables the automation of grading histological tissue engineered cartilage images for quality control standardization. Osteoarthr. Cartil..

[72] Ponce M.C., Zorzi A.R., Miranda J.B.D., Amstalden E.M.I. (2018). Proposal for a new histological scoring System for cartilage repair. Clinics.

[73] Ponce M.C., Zorzi A.R., de Miranda J.B., Amstalden E.M.I. (2018). Proposal for a new histological scoring system for cartilage repair. Clinics.

[74] Audouard E., Rousselot L., Folcher M., Cartier N., Piguet F. (2021). Optimized protocol for subcutaneous implantation of encapsulated cells device and evaluation of biocompatibility. Front. Bioeng. Biotechnol..

[75] Steinert A.F., Ghivizzani S.C., Rethwilm A., Tuan R.S., Evans C.H., Nöth U. (2007). Major biological obstacles for persistent cell-based regeneration of articular cartilage. Arthritis. Res. Ther..

[76] Calabrese G., Gulino R., Giuffrida R., Forte S., Figallo E., Fabbi C., Salvatorelli L., Memeo L., Gulisano M., Parenti R. (2017). In vivo evaluation of biocompatibility and chondrogenic potential of a cell-free collagen-based scaffold. Front. Physiol..

[77] Zheng R., Wang X., Xue J., Yao L., Wu G., Yi B., Hou M., Xu H., Zhang R., Chen J., Shen Z., Liu Y., Zhou G. (2021). Regeneration of subcutaneous cartilage in a swine model using autologous auricular chondrocytes and electrospun nanofiber membranes under conditions of varying Gelatin/PCL ratios. Front. Bioeng. Biotechnol..

[78] Chiesa-Estomba C.M., Aiastui A., González-Fernández I., Hernáez-Moya R., Rodiño C., Delgado A., Garces J.P., Paredes-Puente J., Aldazabal J., Altuna X., Izeta A. (2021). Three-dimensional bioprinting scaffolding for nasal cartilage defects: a systematic review. Tissue Eng Regen Med.

[79] Dupret-Bories A., Chabrillac E., Poissonnet V., Nolens G., Henriet V., Bouakaz I., Sarini J., Grossin D., Riviere L.-D., Vergez S., Vairel B. (2023). Total Nasal Reconstruction in Irradiated Tissue, a New Hope.

[80] Wiggenhauser P.S., Schwarz S., Rotter N. (2018). The distribution patterns of COMP and matrilin-3 in septal, alar and triangular cartilages of the human nose. Histochem. Cell Biol..

[81] Kobayashi S., Takebe T., Inui M., Iwai S., Kan H., Zheng Y.-W., Maegawa J., Taniguchi H. (2011).

[82] Gvaramia D., Kern J., Jakob Y., Zenobi-Wong M., Rotter N. (2022). Regenerative potential of perichondrium: a tissue engineering perspective. Tissue Engineering Part B: Reviews.

[83] Izadyari Aghmiuni A., Heidari Keshel S., Sefat F., AkbarzadehKhiyavi A. (2021). Fabrication of 3D hybrid scaffold by combination technique of electrospinning-like and freeze-drying to create mechanotransduction signals and mimic extracellular matrix function of skin. Mater. Sci. Eng. C..

[84] Tallitsch R. Guastaferri (2021). Histology: an Identification Manual, 2^e^ Édition.

[85] Loqman M., Bush P., Farquharson C., Hall A., Centre for Integrative Physiology, School of Biomedical Sciences, University of Edinburgh, Hugh Robson Building, George Square, Edinburgh EH8 9XD, Scotland, UK (2010). A cell shrinkage artefact in growth plate chondrocytes with common fixative solutions: importance of fixative osmolarity for maintaining morphology. eCM.

[86] Hunziker E.B., Lippuner K., Shintani N. (2014). How best to preserve and reveal the structural intricacies of cartilaginous tissue. Matrix Biol..

[87] Pauwels E., Van Loo D., Cornillie P., Brabant L., Van Hoorebeke L. (2013). An exploratory study of contrast agents for soft tissue visualization by means of high resolution x‐ray computed tomography imaging. J. Microsc..

[88] Quintarelli G., Zito R., Cifonelli J.A. (1971). On phosphotungstic acid staining. J. Histochem. Cytochem..

[89] Sutter S., Todorov A., Ismail T., Haumer A., Fulco I., Schulz G., Scherberich A., Kaempfen A., Martin I., Schaefer D.J. (2017). Contrast-enhanced microtomographic characterisation of vessels in native bone and engineered vascularised grafts using ink-gelatin perfusion and phosphotungstic acid. Contrast. Media. Mol. Imaging..

[90] Karhula S.S., Finnilä M.A., Lammi M.J., Ylärinne J.H., Kauppinen S., Rieppo L., Pritzker K.P.H., Nieminen H.J., Saarakkala S. (2017). Effects of articular cartilage constituents on phosphotungstic acid enhanced micro-computed tomography. PLoS One.

[91] Dullin C., Ufartes R., Larsson E., Martin S., Lazzarini M., Tromba G., Missbach-Guentner J., Pinkert-Leetsch D., Katschinski D.M., Alves F. (2017). μCT of ex-vivo stained mouse hearts and embryos enables a precise match between 3D virtual histology, classical histology and immunochemistry. PLoS One.

[92] Bommakanti K.K., Iyer J.S., Sagi V., Brown A., Ma X., Gonzales M., Stankovic K.M. (2022). Reversible contrast enhancement for visualization of human temporal bones using micro computed tomography. Front. Surg..

[93] Buytaert J., Goyens J., De Greef D., Aerts P., Dirckx J. (2014). Volume shrinkage of bone, brain and muscle tissue in sample preparation for Micro-CT and light sheet fluorescence microscopy (LSFM). Microsc. Microanal..

[94] Salmon P. (2009).

