# Supplementary material for: A proof-of-concept study of an albumin-based bilayered scaffold for cartilage regeneration
Source: Mater Today Bio. 2026 May 2;38:103193. doi: 10.1016/j.mtbio.2026.103193 (PMC13196399; doi:10.1016/j.mtbio.2026.103193)
Supplement: Multimedia component 1 [file mmc1.docx]

**A Proof-of-Concept Study of an Albumin-Based Bilayered Scaffold For Cartilage Regeneration**

Christelle Bertsch, Florent Colin, Eya Aloui, Julien Graff, Maria Cristina Antal, Sabine Kuchler-Bopp, Adrien Moya, Romy Marek, Sven Zaugg, Eric Mathieu, Claire Thibault, Christian Debry, Jordan Beurton, Bernard Senger, Benoit Frisch, Michael de Wild, Arnaud Scherberich, Philippe Lavalle*, Léa Fath

Supporting Information


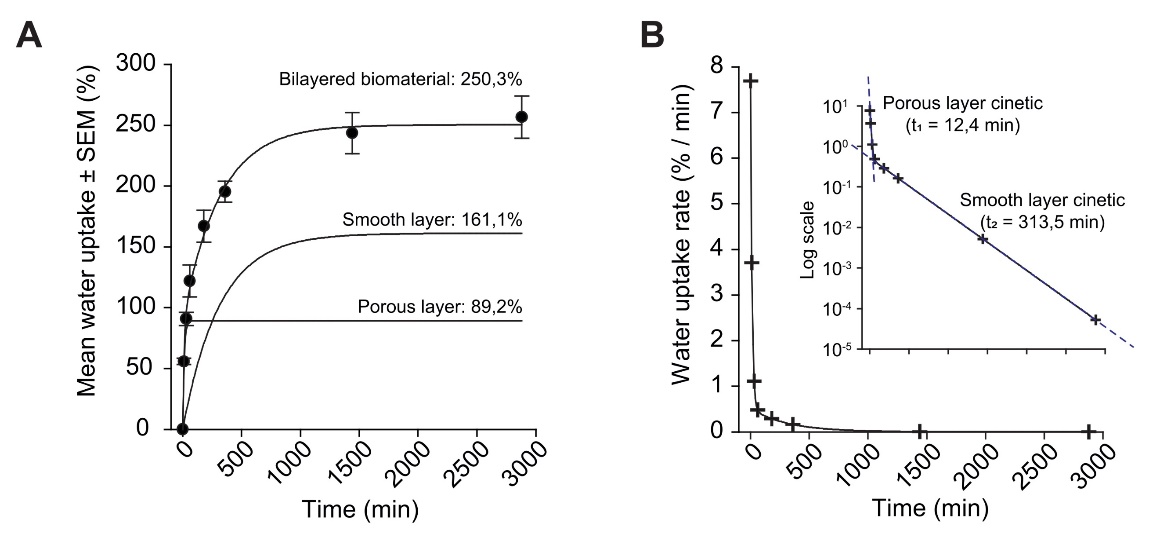


**Figure S1: Quantitative analysis and modeling of scaffold water uptake dynamics.** (A) Experimental measurement of water uptake over time, represented by discrete data points at t=10 min, 30 min, 1h, 6h, 24h, and 48h (mean±SEM, n=4). Superimposed solid lines correspond to the best-fit curves derived from a bi-exponential model, accounting for two kinetically distinct absorption phases. The model outputs are shown separately for the porous layer (bottom: 89.2% of the uptake), the smooth layer (middle: 161.1% of the uptake), and the full scaffold (top: 250.3% of water uptake). (B) First derivative of the fitted water uptake curve (top one, in A), indicating the water absorption rate over time (% per minute). Both linear and logarithmic (top right) scales are presented to highlight the biphasic kinetic behavior. t₁ and t₂ represent the characteristic absorption times of the porous and smooth layers, respectively. These values correspond to the time points at which each layer reaches approximately 63.21% of its total water uptake capacity. SEM: standard error of the mean.


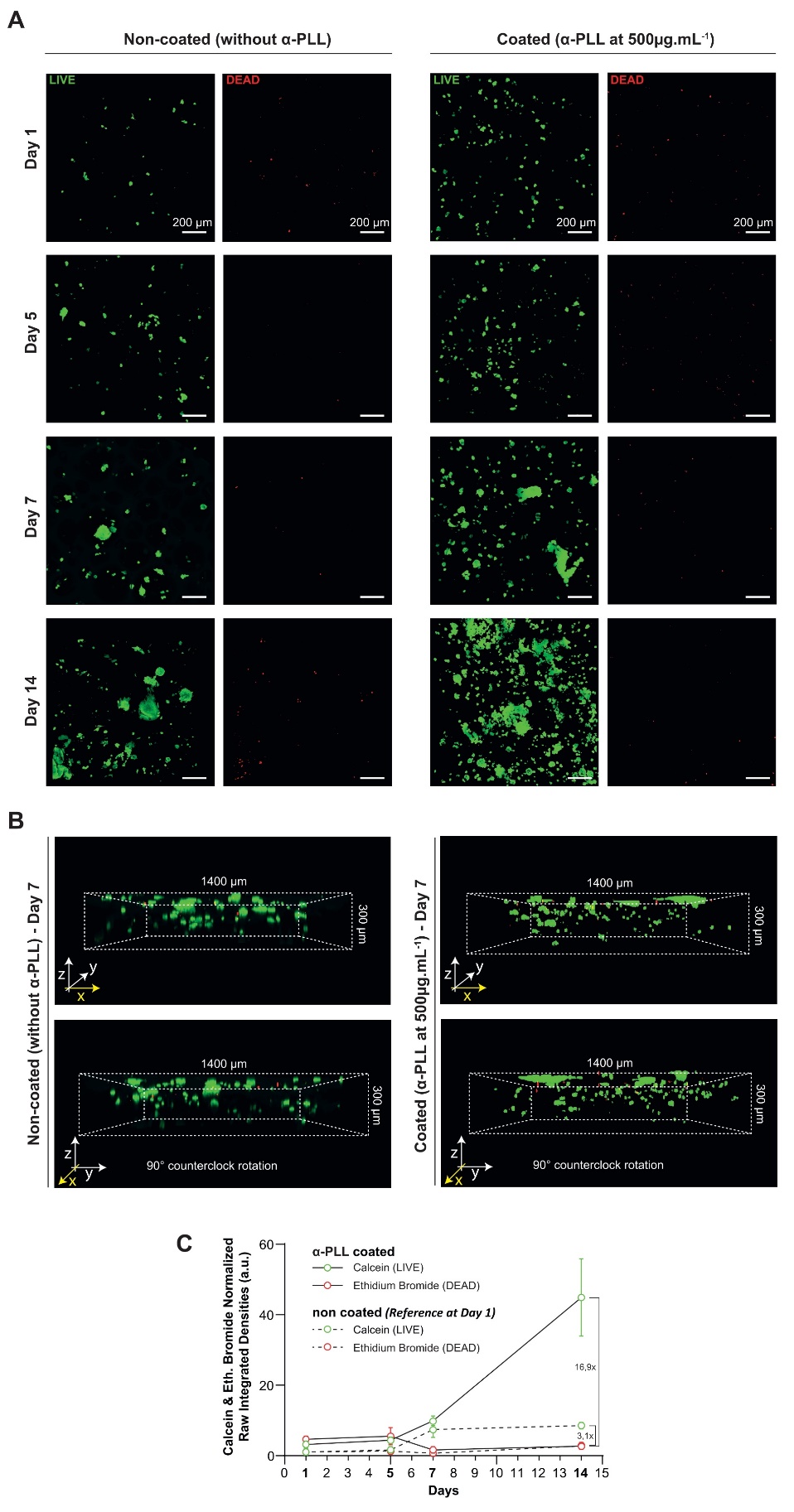


**Figure S2. Assessment of cell viability within the porous layer of bilayered albumin-based scaffolds using LIVE/DEAD staining.** (A) Representative confocal Z-stack projection images of the porous scaffold layer on days 1, 5, 7, and 14, showing the viability of BALB/c-3T3 cells. Viable cells are labeled with calcein (green), while non-viable cells are stained with ethidium bromide (red). (B) Three-dimensional reconstruction of the Z-stack acquired on day 7, shown in frontal view and after a 90° leftward rotation, illustrating the cellular distribution within the scaffold’s porous layer. (C) Quantitative analysis of cell viability based on the normalized raw integrated density of the calcein (green) and ethidium bromide (red) fluorescence signals. Fluorescence values were normalized to the non-coated scaffold condition on day 1. Data are expressed as mean ± standard error of the mean, with five to nine randomly selected regions analyzed per condition across time points (days 1, 5, 7, and 14), in both α-poly-L-lysine (α-PLL)-coated and non-coated scaffolds. (ntot = 4).


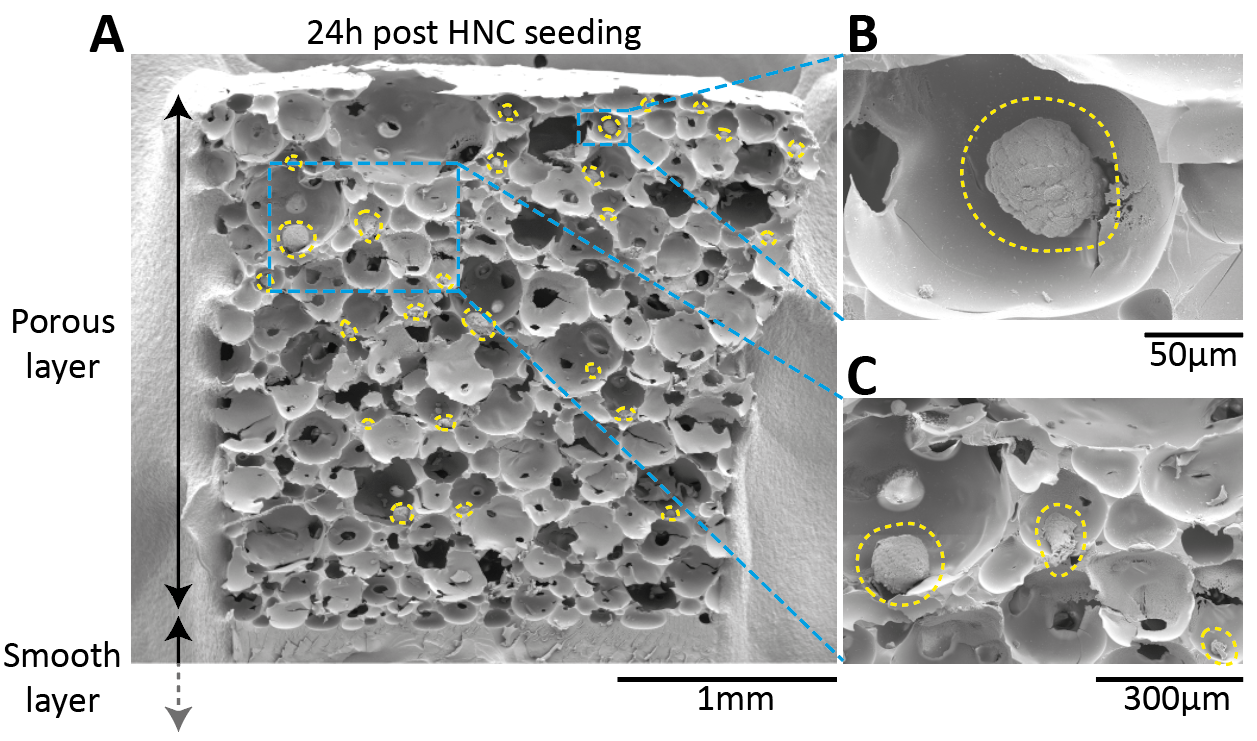


Figure S3. Early HNC infiltration and colonization within the porous layer of the bilayered scaffold. A) Cross-sectional scanning electron microscopy (SEM) images of the scaffold’s porous layer on day 1. HNCs appeared spherical and formed clusters with an uneven distribution but presence throughout the entire thickness of the porous layer. HNCs are represented by yellow dashed circles. (B, C) Cross-sectional SEM images with magnification.


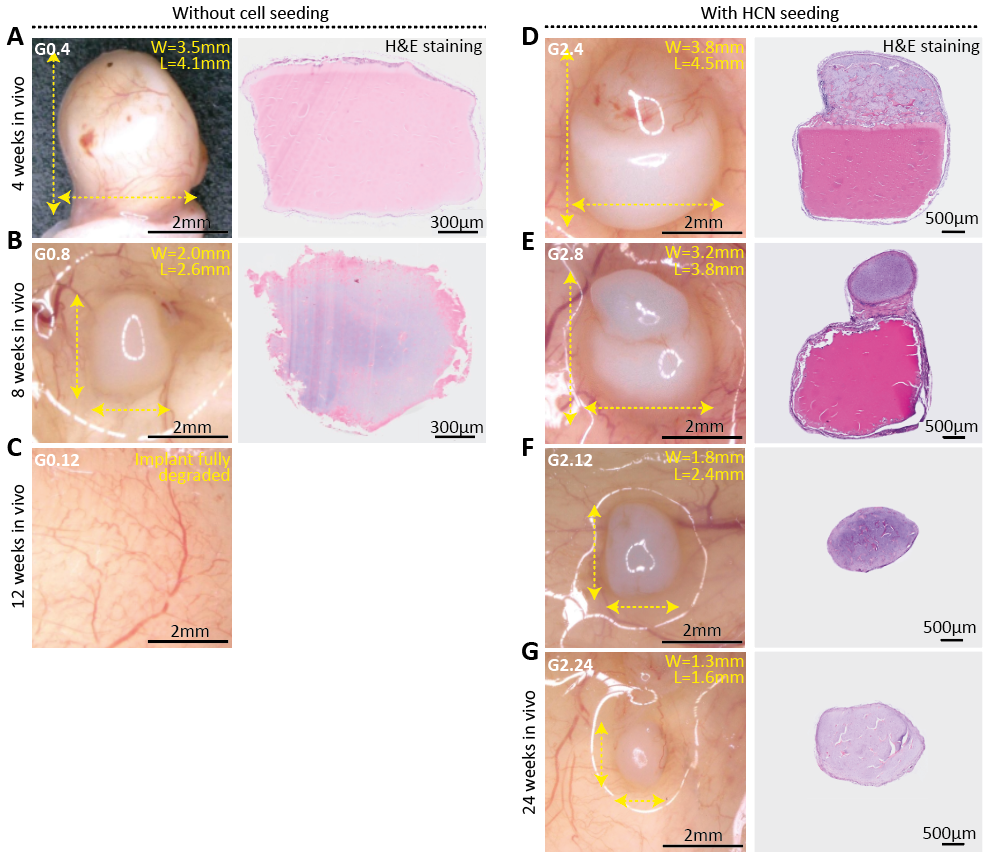


**Figure S4. *In vivo* progressive bilayered scaffold degradation and cartilage-like tissue formation with and without HNC seeding after 4, 8, 12 and 24 weeks of subcutaneous implantation in nude mice.** (A–G) Macroscopic images of the bilayered scaffold and *in vivo* cartilage-like tissue formation, with corresponding histological analysis of explants using hematoxylin/eosin (H&E) staining to assess tissue morphology. (A) G0.4 (n = 10 mice); (B) G0.8 (n = 5); (C) G0.12 (n = 5); (D) G2.4 (n = 12); (E) G2.8 (n = 7); (F) G2.12 (n = 7); and (G) G2.24 (n = 7). H&E staining could not be performed for G0.12, as implants without cell seeding were fully degraded.


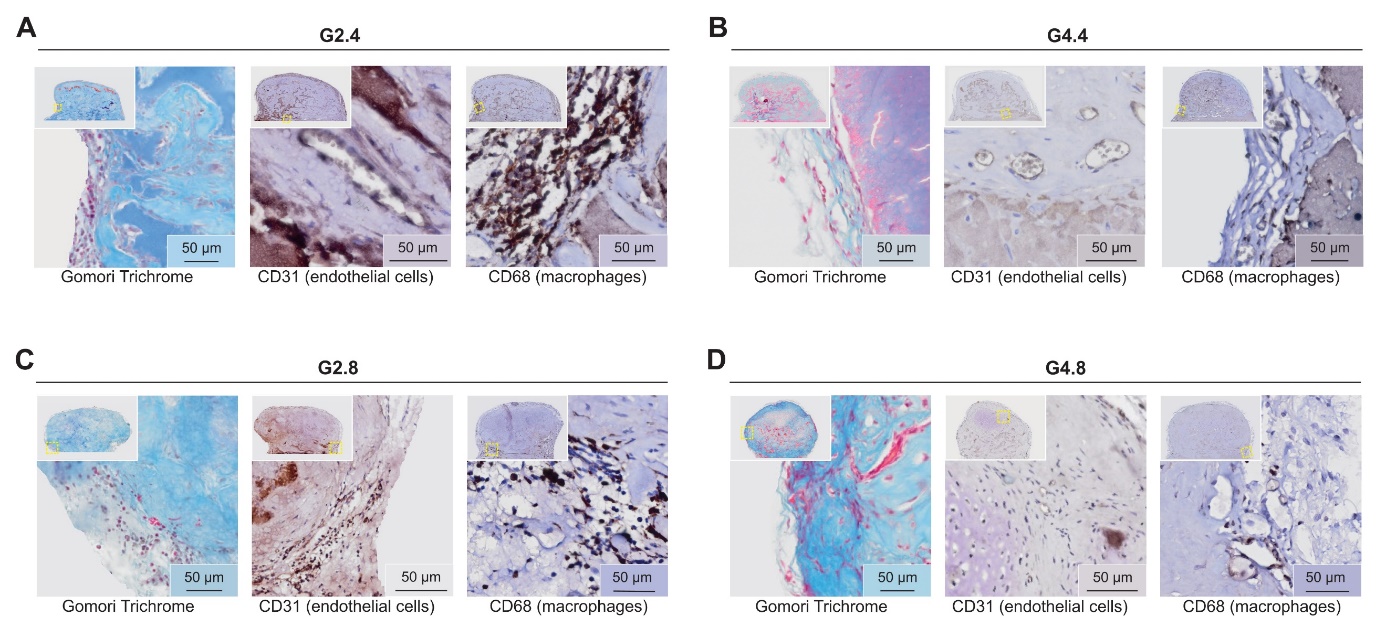


Figure S5. *In vivo* evaluation of the porous layer of the albumin-based scaffold after 2 or 4 weeks of *in vitro* chondrogenic redifferentiation and 4 or 8 weeks of subcutaneous implantation in nude mice (G2.4, n = 12 mice / G4.4, n = 12 mice / G2.8, n = 7 mice and G4.8, n = 7 mice). Histological sections from groups (A) G2.4, (B) G4.4, (C) G2.8, and (D) G4.8 were stained using Gomori’s trichrome to assess general tissue morphology and matrix organization. Immunohistochemical stainings were performed for CD31 (a marker of endothelial cells) and CD68 (a marker of macrophages) to evaluate neovascularization and host immune cell infiltration, respectively. Each image includes an inset showing a zoomed-out overview of the sample (about 23× for both G2.4 and G4.4, and about 18× for both G2.8 and G4.8).


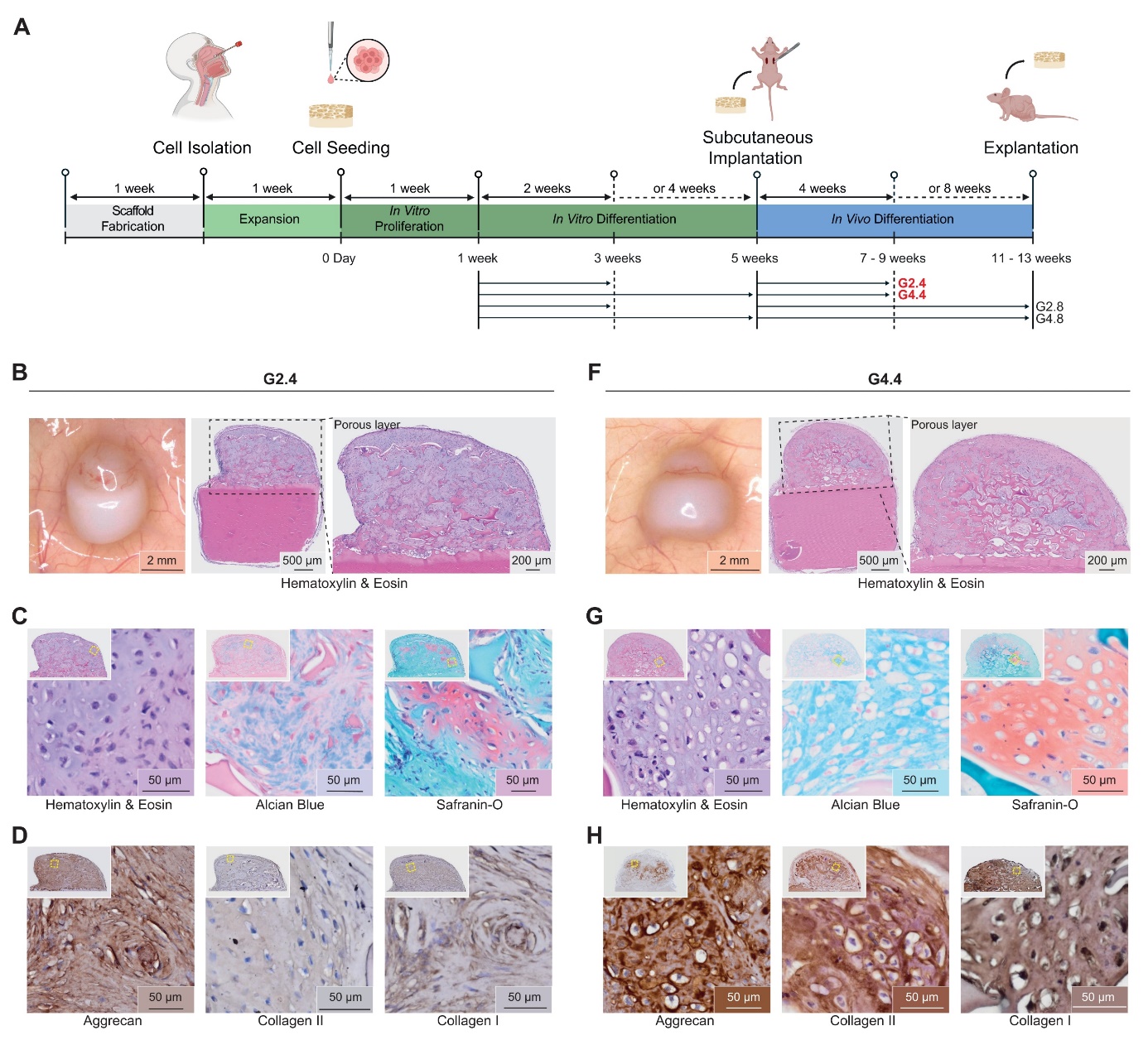


**Figure S6. *In vivo* evaluation of the porous layer of the albumin-based scaffold after 2 or 4 weeks of *in vitro* chondrogenic redifferentiation and 4 weeks of subcutaneous implantation in nude mice (G2.4, n = 12 mice and G4.4, n = 12 mice).** (A) Overview of the experimental design. Created with BioRender.com and finalized in Adobe Illustrator. (B and F) Macroscopic images of the cartilage-like tissue that formed on the scaffold’s porous layer after 4 weeks of implantation. (C and G) Histological analysis of explants using hematoxylin/eosin (H&E), Alcian **B**lue (AB), and **S**afranin-O (SO) stainings to evaluate tissue morphology and glycosaminoglycan content. (D and H) Immunohistochemical stainings for cartilage-specific aggrecan, collagen type II, and collagen type I markers. Positive immunoreactivity is visualized using the brown diaminobenzidine tetrahydrochloride chromogen. Each histological image includes an inset showing a zoomed-out overview of the sample (about 22× for both G2.4 and G4.4).


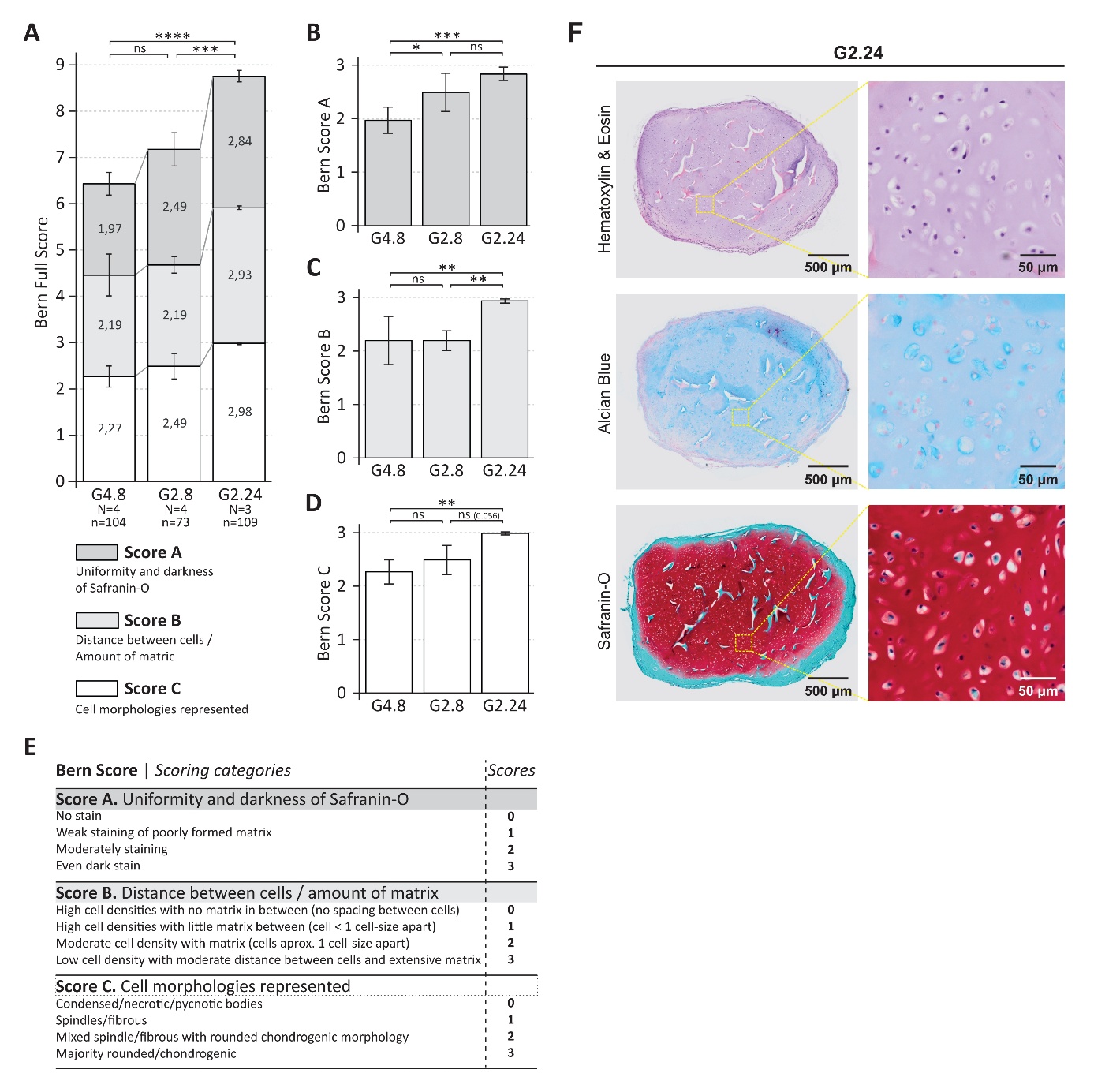


Figure S7. Bern Score for evaluation of the *in vivo* cartilage regeneration for after 2 or 4 weeks of *in vitro* chondrogenic redifferentiation and 8 or 24 weeks of subcutaneous implantation in nude mice (G4.8, n = 7 mice / G2.8, n = 7 mice and G2.24, n = 7 mice). (A) Compilation of the results and statistical analyses. (B-D) Comparison of Bern score A, B and C, respectively, with statistical analyses. (E) Bern scoring categories. N corresponds to the number of independent experiments. n corresponds to the number of 250 × 250 µm regions of interest (ROIs) evaluated twice by two different experimenters. (F) Histological analysis of G2.24 explants using hematoxylin/eosin (H&E), Alcian Blue (AB), and Safranin-O (SO) stainings to evaluate tissue morphology and glycosaminoglycan content.


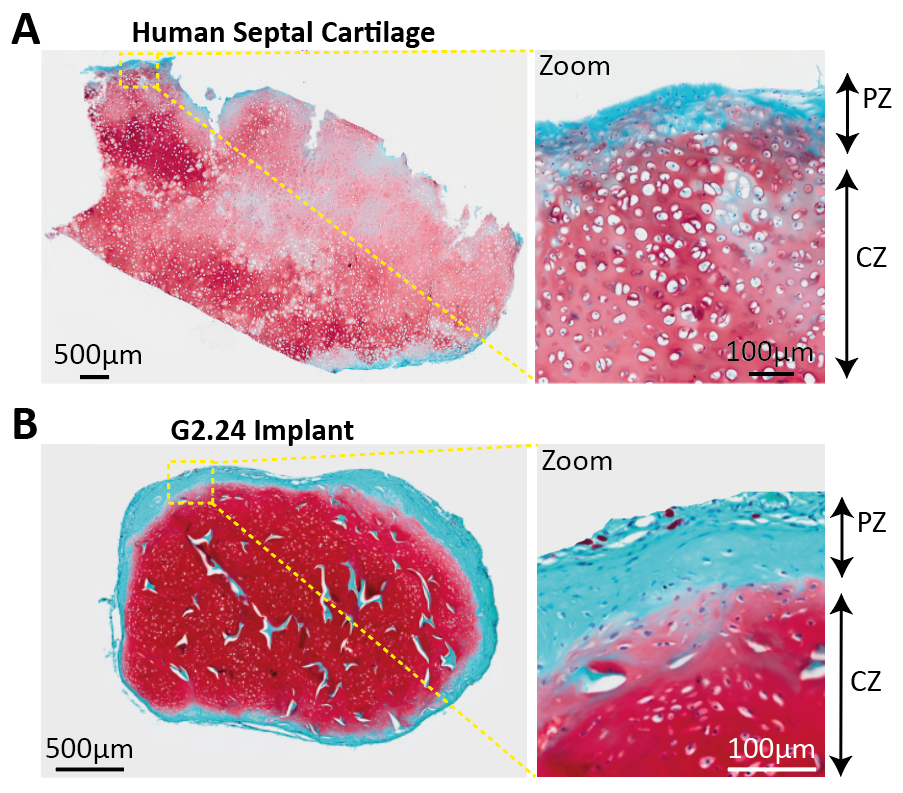


**Figure S8. Comparison of zonal architecture (PZ and CZ) between (A) human native septal cartilage and (B) G2.24 (n=7 mice).** (B) Histological analysis of G2.24 explants using Safranin-O (SO) staining to assess glycosaminoglycan content and identify both zones. Representative histology images of human native septal cartilage are selected from three independent experiments.
